# Adaptive Cognitive Intervention Architecture: An Exploratory Computational Framework for Precision Reading Comprehension in Higher Education

**DOI:** 10.3390/jintelligence14070143

**Published:** 2026-07-08

**Authors:** Teófilo Félix Valentín Melgarejo, Gastón Jeremías Oscátegui Nájera, Dora Marina Hachoque Aguirre, Ulises Espinoza Apolinario, Isela Silvia Cruz Quinto, Fidel Alberto García Yale, Liz Ketty Bernaldo Faustino, Clodoaldo Ramos Pando, Josué Chacón Leandro, Alexandra Rivas Meza, Pablo Lenin La Madrid Vivar, José Rovino Alvarez Lopez, Pablo Lolo Valentín Melgarejo, Flaviano Armando Zenteno Ruiz

**Affiliations:** 1 School of Secondary Education, Faculty of Educational Sciences, Daniel Alcides Carrión National University, Cerro de Pasco 19001, Peru; uespinozaa@undac.edu.pe (U.E.A.); lbernaldof@undac.edu.pe (L.K.B.F.); cramos@undac.edu.pe (C.R.P.); plamadridv@undac.edu.pe (P.L.L.M.V.); jalvarez@undac.edu.pe (J.R.A.L.); fzentenor@undac.edu.pe (F.A.Z.R.); 2 School of Primary Education, Faculty of Educational Sciences, Daniel Alcides Carrión National University, Cerro de Pasco 19001, Peru; goscateguin@undac.edu.pe (G.J.O.N.); scruzq@undac.edu.pe (I.S.C.Q.); jchaconl@undac.edu.pe (J.C.L.); pvalentinm@undac.edu.pe (P.L.V.M.); 3 School of Secondary Education, Faculty of Educational Sciences, Enrique Guzmán y Valle National University, La Cantuta, Lima 15472, Peru; dhachoque@une.edu.pe; 4 School of Primary Education, Faculty of Educational Sciences, Herminio Valdizan National University, Pillco Marca, Huánuco 10001, Peru; fgarcia@unheval.edu.pe; 5 School of Primary Education, Faculty of Educational Sciences, Juan Santos Atahualpa National Intercultural University of the Central Jungle, Junín 12000, Peru; arivas@uniscjsa.edu.pe

**Keywords:** reading comprehension, metacognitive learning, explainable artificial intelligence, precision education, adaptive learning analytics

## Abstract

Reading comprehension is a critical cognitive competency in higher education, although learners demonstrate substantial variability in responsiveness to metacognitive instructional interventions. The study focused on individual cognitive-response processes within the framework of the adaptive metacognitive reading system, which was realized through precision-learning architecture, which integrates latent learner-response phenotyping, explainable machine learning, Markov transition analysis, Bayesian adaptive inference, and reinforcement-learning optimization. The study employed a quasi-experimental longitudinal design involving an eight-week structured metacognitive reading intervention delivered through planning, monitoring, evaluation, strategic flexibility, and reading self-regulation activities. The psychometric analyses demonstrated satisfactory reliability of the adapted Metacognitive Awareness Inventory (MAI), with Cronbach’s *α* ranging from 0.83 to 0.89. A latent-profile model revealed significant heterogeneity of learner-response patterns among learners, with four learner-response phenotypes: High Responders, Strategic Improvers, Monitoring-Dependent Learners, and Low Responders. Explainable machine-learning models performed well in predicting individualized comprehension gains, with the model with the highest predictive accuracy being XGBoost (R^2^ = 0.61). Markov transition modeling identified exploratory learner-state redistribution patterns following the intervention. Bayesian adaptive inference and reinforcement-learning optimization were subsequently conducted as post hoc simulation procedures to estimate hypothetical adaptive instructional calibration scenarios rather than as real-time instructional decision systems. Overall, the proposed Adaptive Cognitive Intervention Architecture (ACIA) should be interpreted as an exploratory computational framework for modeling learner heterogeneity, predicting comprehension gains, and simulating post hoc computational optimization in higher-education learning environments.

## 1. Introduction

Comprehension skills are among the most fundamental cognitive skills in higher education and have a direct effect on analytical thinking, academic performance, knowledge integration and the ability to engage in lifelong learning (Javorčíková et al., 2021). Modern higher-education environments continuously require learners to process large volumes of information, critically evaluate complex arguments, integrate knowledge across disciplines, and regulate their cognitive strategies during academic reading. Although instructional strategies have advanced considerably, many university students continue to experience difficulties with reading-comprehension monitoring, inference-making, strategic assessment and self-regulation during reading activities, resulting in differences in academic achievement and greater variability in learning outcomes. These difficulties become more prominent in educational settings where learning processes are cognitively demanding, learning strategies are fragmented, metacognitive awareness is limited, and self-regulation during complex reading tasks is insufficient (Flavell, 1979; Zimmerman, 2002; Schraw & Dennison, 1994; Vardanjani et al., 2025).

Metacognition has become one of the most significant theoretical frameworks accountings for inter-individual variance in learning efficiency, comprehension performance and adaptive cognitive regulation. Metacognition is defined as the ability to plan, monitor, evaluate and manage cognitive processes during learning activities (Efklides, 2006). Research has consistently shown that students with stronger metacognitive regulation skills demonstrate better reading comprehension, greater strategic flexibility, enhanced problem-solving ability, and higher academic persistence (Pintrich, 2000; Schraw et al., 2006; Hung & Loh, 2021; İdawati et al., 2020). Planning, monitoring, and evaluation enable learners to organize comprehension processes, identify inconsistencies, regulate effort, and adapt their reading strategies according to contextual demands. Consequently, metacognitive instruction has become increasingly important in educational interventions designed to enhance reading comprehension and self-regulated learning (SRL) skills.

Although previous studies have demonstrated the benefits of metacognitive interventions for reading comprehension, much of the existing literature has relied on linear statistical models and variable-centered analytical approaches that implicitly assume learner homogeneity (Dignath & Büttner, 2008; Donker et al., 2014). Consequently, the average intervention effect reported by traditional regression-based models may overlook substantial inter-individual differences in cognitive responsiveness, metacognitive readiness, and self-regulatory organization. Both foundational and contemporary studies suggest that learners exhibit heterogeneous developmental responses to instruction, with complex interactions among monitoring, evaluation, strategic flexibility, and contextual learning conditions influencing individual learning trajectories (Azevedo & Cromley, 2004; Greene & Azevedo, 2007; Panadero, 2017; Theobald, 2021). Consequently, conventional analytical approaches may be insufficient to capture latent learner-response phenotypes, learner-state redistribution patterns, and individualized learner-response patterns underlying improvements in reading comprehension.

With recent developments in learning analytics, explainable artificial intelligence (XAI), Bayesian adaptive modeling, and precision-learning systems, new opportunities have emerged to study multidimensional cognitive heterogeneity in educational contexts. Explainable machine-learning approaches such as SHAP (Shapley Additive Explanations) can be used to estimate the nonlinear contributions of predictors and interaction topologies while maintaining the interpretability of predictive models (Lundberg & Lee, 2017). Likewise, person-centered methods such as latent profile analysis and adaptive clustering enable the identification of hidden learner-response phenotypes that may not be detected using conventional variable-centered methods (Howard & Hoffman, 2018; Conradt et al., 2025). Bayesian adaptive systems and reinforcement-learning architectures have the potential to model hypothetical adaptive instructional scenarios, learner-state redistribution patterns, and individualized learning conditions in heterogeneous learner populations (Luckin et al., 2016; Baker & Inventado, 2014; Hu et al., 2025). Despite these methodological advancements, research integrating explainable machine learning, latent cognitive phenotyping, nonlinear response-surface analysis and reinforcement-learning optimization within metacognitive reading interventions remain limited in the current educational literature.

For clarity, several technical concepts used in this study are briefly defined. Learner-response phenotyping refers to the identification of groups of learners who exhibit similar metacognitive characteristics. Explainable machine-learning modeling refers to predictive algorithms whose decision-making processes can be interpreted. Nonlinear cognitive landscape analysis refers to the visualization of relationships between multiple cognitive factors and learning outcomes. Markov transition analysis examines how learners redistribute between categories across observation periods. Bayesian adaptive inference estimates probabilistic relationships while explicitly quantifying uncertainty. Reinforcement-learning optimization is a simulation-based approach that explores hypothetical instructional policies by maximizing long-term rewards under different learner-state conditions.

Furthermore, previous studies on metacognition have tended to emphasize only pretest-posttest comparisons and have rarely investigated learner-state redistribution patterns and nonlinear changes in learner-state membership following instructional interventions. Existing educational research has seldom examined whether learners exhibit stable learner-response phenotypes, whether intervention responsiveness can be described in terms of discrete learner-response patterns, or whether adaptive instructional architectures can support individualized learning patterns over time. Likewise, few studies have integrated psychometric, computational, Bayesian, and explainable-AI approaches into a single precision-metacognitive framework capable of prediction, interpretation, adaptive simulation, and intervention optimization. This methodological fragmentation limits our understanding of how multidimensional regulatory systems influence improvements in reading comprehension following instructional intervention.

This study aims to overcome these theoretical and methodological shortcomings by developing an integrated Adaptive Cognitive Intervention Architecture (ACIA) that integrates latent learner-response phenotyping, explainable machine-learning modeling, nonlinear cognitive landscape analysis, Markov transition analysis, Bayesian adaptive inference, and reinforcement-learning optimization within a metacognitive reading-intervention framework. The current research conceptualizes comprehension development as a nonlinear, probabilistic, and heterogeneous process resulting from the coordinated interactions among regulation, monitoring, evaluation, strategy flexibility, and metacognitive knowledge. The study also explores learner-state redistribution patterns and simulation-based reinforcement-learning structures to investigate how learner states may change following exposure to the intervention.

From a pedagogical perspective, effective metacognitive instruction involves explicitly teaching students to plan comprehension strategies, monitor their understanding during reading, evaluate their comprehension, and adjust their regulatory behavior when comprehension difficulties arise. These self-regulated learning environments should be technologically supported and pedagogically designed to incorporate scaffolding that accommodates students with different levels of readiness and metacognitive orientations. Thus, hypothetical adaptive instructional scenarios should be understood not merely as computational systems but as pedagogically informed frameworks for providing cognitive support and strategic learning in higher-education settings.

The study presents a person-centered explainable-AI framework that supports the identification of latent learner-response phenotypes associated with different levels of reading-comprehension gain. Second, the study applies SHAP-based nonlinear interaction-topology analysis to examine the multidimensional metacognitive contribution structures underlying individualized intervention responsiveness. Third, Markov transition analysis and entropy-based state diagnostics are used to explore learner-state redistribution patterns between observation periods. Fourth, hypothetical instructional calibration scenarios and prospective instructional recommendations are investigated through simulation-based Bayesian adaptive inference and reinforcement-learning optimization. Finally, the study introduces the ACIA as an exploratory computational framework that integrates latent learner-response phenotyping, predictive modeling, and post hoc simulation-based optimization rather than as a real-time adaptive instructional system implemented during classroom instruction. The following research questions guided the present study:

RQ1. Can distinct latent learner-response phenotypes be identified among undergraduate students following a structured metacognitive reading intervention?

RQ2. Which baseline metacognitive dimensions (planning, monitoring, evaluation, metacognitive knowledge, and strategy flexibility) exhibit the strongest nonlinear associations with individualized reading-comprehension gains?

RQ3. How are learner-state redistribution patterns distributed between pre-intervention and post-intervention observations?

RQ4. Can explainable machine-learning and Bayesian modeling accurately predict heterogeneous learner-response patterns?

RQ5. Can reinforcement-learning simulations generate hypothetical precision-oriented instructional recommendations under different learner-state conditions?

## 2. Materials and Methods

### 2.1. Study Design and Analytical Framework

The study employed a single-group quasi-experimental pretest-posttest longitudinal design without a control group. All 180 participants received the same structured metacognitive reading intervention, and subsequent analyses focused on learner heterogeneity, predictive modeling, and exploratory computational simulations.

Following data collection, several analytical procedures were performed, including psychometric validation, latent learner-response phenotyping, explainable machine-learning modeling, Markov transition analysis, Bayesian adaptive inference, and reinforcement-learning simulations. These analytical procedures should not be interpreted as components of the study design but rather as post hoc methods used to evaluate learner heterogeneity, predictive performance, and learner-response patterns.

It is important to distinguish between the instructional intervention implemented in the classroom and the computational architecture applied after data collection. The classroom component consisted of a structured eight-week metacognitive reading intervention delivered by instructors through planning activities, monitoring prompts, evaluative reflection, strategic flexibility tasks, and corrective feedback. The ACIA components, including latent learner-response phenotyping, explainable machine learning, Markov transition modeling, Bayesian adaptive inference, and reinforcement-learning optimization, were not used to guide real-time instructional decisions during the intervention (Table 1). These components were applied post hoc as analytical and simulation-based procedures to model learner heterogeneity, estimate learner-state redistribution patterns, predict comprehension gains, and explore hypothetical adaptive instructional scenarios.

### 2.2. Participants and Sampling Procedure

Participants were selected using proportionate stratified probability sampling, with academic year serving as the stratification variable to preserve the natural distribution of eligible students. An initial pool of 245 undergraduate students was screened for eligibility. After applying the inclusion and exclusion criteria, 180 students were retained for participation. Thirty-one students did not meet the eligibility criteria, 18 declined participations, and 16 were excluded because of incomplete baseline assessments. Consequently, the final analytical sample consisted of 180 undergraduate students. Because the study employed a single-group quasi-experimental design, all participants received the same structured metacognitive intervention, and no control group was included. Differences in academic-year percentages reflected the underlying composition of the eligible student population rather than equal allocation across strata.

Of this total, 96 of the students were female (53.3%) and 84 students were male (46.7%). Most participants were between 19 and 20 years of age (43.3%), followed by students aged 17–18 years (27.2%), 21–22 years (22.8%), and ≥23 years (6.7%). The distribution of students by their academic level was 13.3% (4th year), 23.9% (3rd year), 33.9% (2nd year), and 28.9% (1st year). Other demographic, academic and metacognitive-background characteristics are summarized in Supplementary Table S1.

The inclusion criteria were operationalized as follows: (i) active enrollment was verified through official university enrollment records and course registration lists for the academic semester; (ii) regular engagement in academic reading activities was defined as enrollment in reading-intensive coursework and documented completion of weekly academic reading assignments before the intervention; (iii) intervention participation required attendance in at least 70% of the scheduled instructional sessions; and (iv) analytical inclusion required completion of both baseline and post-intervention assessments. Students who did not meet these criteria declined participation, or had incomplete baseline data were excluded.

### 2.3. Study Location

The study was carried out at the Universidad Nacional Daniel Alcides Carrión, in the undergraduate program of Communication and Literature, in the Faculty of Education of Cerro de Pasco, Peru. The participants were undergraduate students in the academic semester of 2025, who regularly participated in reading-intensive academic activities.

### 2.4. Structured Metacognitive Reading Intervention

The intervention was primarily designed according to Zimmerman’s Self-Regulated Learning framework (Zimmerman, 2000, 2002), which conceptualizes learning as a cyclical process involving forethought, performance monitoring, and self-reflection phases. Flavell’s metacognitive theory (Flavell, 1979) and Pintrich’s self-regulated learning framework (Pintrich, 2000) were also incorporated to strengthen the development of planning, monitoring, evaluation, strategic flexibility, and self-regulatory competencies throughout the intervention.

The intervention involved an 8-week adaptive metacognitive instructional program that explicitly taught planning, monitoring, evaluation, metacognitive knowledge and strategic flexibility to boost reading comprehension. Instructional architecture incorporated elements of self-regulated learning, guided reflection, adaptive scaffolding, evaluative feedback, and individualized cognitive support.

The instructional session was approximately 90 min long and was carried out twice per week for a total of 16 instructional sessions during the study period. Therefore, the complete intervention duration corresponded to 8 consecutive instructional weeks. Sessions featured strategically guided metacognitive questioning, monitoring checklists, evaluative reflection journals, adaptive comprehension scaffolding, collaborative analytical-reading activities, and individualized corrective feedback mechanisms. In reading performance, students gradually encountered increasingly difficult reading comprehension tasks that involved making inferences, making strategic adaptations, making evaluative synthesis, and coordinating regulatory coordination.

#### Pedagogical Structure and Metacognitive Instructional Procedures

The intervention was based on the concepts of self-regulated learning (SRL), metacognitive instruction, guided cognitive apprenticeship, and adaptive pedagogical scaffolding. Planning, monitoring, evaluation and strategic flexibility were clearly taught during authentic academic reading activities.

Instructional sessions were designed to employ a metacognitive sequence of pedagogical phases: (i) activation and planning, (ii) guided reading and monitoring, (iii) collaborative analytical interpretation, (iv) evaluative reflection, and (v) adaptive feedback and strategic adjustment.

In the planning phase, students set up reading goals, brought prior metacognitive knowledge, forecasted text structure, anticipated task difficulty and chose comprehension strategies prior to reading. Strategies for strategic planning were modeled by instructors through think-aloud and metacognitive questions (e.g., What is my goal while reading this section? And which strategy will help me understand complex arguments?).

In guided reading activities, students completed metacognitive checklists, annotations, pauses for checking comprehension, and inference tracking activities. The students were encouraged to identify comprehension breakdowns, ambiguity, cognitive overload and inconsistencies in interpretation when reading academic texts.

Students were involved in collaborative analytical-reading activities that involved comparing interpretations, inferring, drawing together arguments within paragraphs, and evaluating evidence in small-group discussions. The activities were designed to support strategic flexibility, evaluative reasoning and adaptive comprehension regulation.

Structured reflective journals were used during the evaluative-reflection phase and students were asked to record comprehension problems, effective strategies, unsuccessful strategies and corrective adaptations after each session. Reflection questions led students to assess the effectiveness of strategies and their willingness to change their approach to reading in the future.

The instructor primarily functioned as a metacognitive facilitator rather than a direct transmitter of information. Instructional support consisted of guided questioning, adaptive scaffolding, individual corrective feedback, monitoring prompts, and a graduated reduction in support based on learner responsiveness and metacognitive readiness.

Instructional differentiation was planned according to learners observed metacognitive readiness during the intervention. Students with less monitoring and evaluative regulation were provided with more scaffolding, less complicated comprehension segmentation, and instructors guided monitoring prompts. Students with higher metacognitive readiness, however, completed more advanced evaluative synthesis tasks and more complex inferential-reading tasks, in which they needed to exert more self-regulated control, and performed independent strategic regulation activities.

Instructional materials included academic passages that required reading to interpret in argumentative, inferential, evaluative, and strategic ways, as well as to integrate and compare multiple perspectives within texts. Within the intervention, the level of reading difficulty gradually escalated to foster adaptive metacognitive regulation and strategic flexibility in cognitively complex situations.

### 2.5. Measurement Instruments

#### 2.5.1. Adapted Metacognitive Awareness Inventory (MAI)

Metacognitive regulation was assessed using an adapted version of the Metacognitive Awareness Inventory (MAI) developed by Schraw and Dennison (1994). The instrument was modified to align with the objectives of reading-comprehension activities implemented in the present study. Five dimensions were assessed: planning, monitoring, evaluation, metacognitive knowledge, and strategy flexibility. Responses were recorded using a five-point Likert scale ranging from 1 (“Strongly disagree”) to 5 (“Strongly agree”).

The original MAI is a standardized instrument widely used to measure metacognitive awareness and self-regulatory learning processes in educational research (Schraw & Dennison, 1994). Because strategy flexibility was not explicitly included in the original instrument, several context-specific items were incorporated to capture learners’ adaptive strategy adjustment during reading-comprehension tasks. The adapted instrument underwent internal consistency evaluation before subsequent analyses.

The psychometric analysis demonstrated good reliability across all dimensions, with Cronbach’s *α* ranging from 0.83 to 0.89 and McDonald’s ω ranging from 0.84 to 0.89, indicating satisfactory internal consistency for multivariate and computational analyses.

#### 2.5.2. Reading-Comprehension Assessment

The reading-comprehension assessment consisted of two parallel forms administered at baseline and post-intervention to minimize testing and memory effects. Each form contained 20 items evaluating literal comprehension, inferential comprehension, analytical reasoning, and information integration skills.

Parallel forms were developed to possess equivalent content coverage and difficulty levels. Comparability was established through expert review, content alignment procedures, and pilot testing before implementation. Scores were expressed as percentages, with higher scores indicating better reading-comprehension performance.

### 2.6. Data Collection Procedure

Data collection was conducted in three sequential phases.

Phase 1: Baseline assessment

Before exposure to the intervention, participants completed demographic questionnaires, the adapted MAI, and a baseline reading-comprehension assessment. Baseline scores for planning, monitoring, evaluation, metacognitive knowledge, strategy flexibility, and reading-comprehension performance were obtained and subsequently incorporated into computational analyses to characterize learner heterogeneity and baseline metacognitive profiles.

Phase 2: Metacognitive intervention

An 8-week metacognitive instructional intervention was implemented in supervised classroom settings. Throughout the intervention period, instructors maintained structured monitoring records to document intervention adherence, classroom participation, strategy use during reading activities and learning progress. These records were used solely to monitor intervention implementation and participant engagement and were not treated as independent outcome variables in the statistical analyses. Intervention adherence, classroom participation, strategy use, and learning progress were continuously monitored and documented. Instructor monitoring records were maintained during each intervention session using standardized checklists to document attendance, participation, strategy use, and learning progress (Supplementary Table S4).

Phase 3: Post-intervention evaluation

Following the intervention, participants completed a reading-comprehension posttest and re-administered the adapted MAI. These post-intervention assessments were used to evaluate changes in metacognitive regulation and reading-comprehension performance and to support subsequent analyses of learner heterogeneity and individualized learner-response patterns.

### 2.7. Statistical and Computational Analysis

Prior to all analyses, missing-data diagnostics were performed. Participants with incomplete baseline assessments were excluded during participant screening; therefore, no missing values were present in the analytical dataset used for computational modeling. All continuous variables were subsequently standardized using z-score transformation before multivariate and machine-learning analyses. The analytical variables used in each computational stage are summarized in Supplementary Table S3.

#### 2.7.1. Psychometric and Descriptive Analysis

Descriptive statistical analyses included means, standard deviations, skewness, kurtosis, Cronbach’s *α*, and McDonald’s ω coefficients. Distributional diagnostics and reliability analyses were performed before multivariate and computational modeling procedures to confirm the psychometric suitability of the analytical variables. Variance inflation factor (VIF) diagnostics further indicated no severe multicollinearity among predictor variables, with all VIF values remaining below the recommended threshold of 3.0.

#### 2.7.2. Latent Learner-Response Phenotype Identification

Latent learner-response phenotypes were identified using Gaussian Mixture Modeling (GMM) applied exclusively to baseline metacognitive variables, including planning, monitoring, evaluation, metacognitive knowledge, and strategy flexibility. Reading-comprehension gain was intentionally excluded from the clustering procedure and was subsequently analyzed as an external post-intervention outcome variable to avoid circular interpretation.

Before clustering, all variables were standardized using z-score transformation. Multiple random starts, full covariance estimation, and log-likelihood stabilization procedures were implemented to improve solution stability and convergence. Silhouette coefficients, Davies–Bouldin indices, Bayesian Information Criterion (BIC), and latent-density topology analyses were used to determine the optimal cluster structure.

The four-cluster solution demonstrated the highest structural stability and interpretability and yielded four learner phenotypes: High Responders, Strategic Improvers, Monitoring-Dependent Learners, and Low Responders. Uniform Manifold Approximation and Projection (UMAP) was subsequently applied to visualize the nonlinear learner-state structure because the data exhibited heterogeneous and nonlinearly organized metacognitive patterns. Extended validation diagnostics are provided in Supplementary Figure S2. For consistency, a single taxonomy was adopted throughout the manuscript. The four learner phenotypes were designated as High Responders, Strategic Improvers, Monitoring-Dependent Learners, and Low Responders, and these labels were used consistently across the text, tables, figures, and Supplementary Materials.

##### Sensitivity Analysis

A sensitivity analysis was conducted to evaluate the robustness of the baseline-only phenotype solution and to confirm that the learner taxonomy was not driven by post-intervention outcomes. Structural stability was examined using silhouette coefficients, Davies–Bouldin indices, Bayesian Information Criterion values, and profile membership agreement. The four-profile architecture remained stable and interpretable, supporting the robustness of the learner-response taxonomy while minimizing the risk of outcome-driven circularity. Sensitivity-analysis results are provided in Supplementary Table S5.

#### 2.7.3. Explainable Machine-Learning Modeling

Individualized comprehension gains after exposure were predicted with explainable machine learning models. The machine learning algorithms used in the computational framework were: Random Forest, XGBoost, LightGBM, and Elastic Net. Model performance was evaluated using R^2^, RMSE, MAE, calibration error, and 10-fold cross-validation. All preprocessing, feature construction, hyperparameter optimization, and SHAP analyses were conducted independently within each training fold to minimize information leakage and overfitting.

Bayesian optimization and grid search were used to perform hyperparameter optimization independently within each cross-validation training fold. The model configurations and model parameters used for optimization are detailed in Supplementary Table S2. To reduce the possibility of information leakage, preprocessing procedures, including z-score standardization and feature construction, were performed exclusively using training data within each fold and subsequently applied to the corresponding validation data. Validation observations were not used during preprocessing, model training, hyperparameter optimization, or interpretability analyses.

Nonlinear predictor importance, interaction density, and individualized contribution topology across metacognitive dimensions were estimated using SHAP (Shapley Additive Explanations) analysis. Extended SHAP interaction diagnostics can be found in Supplementary Figure S3.

The predictive analyses were designed to estimate heterogeneous learner responses and variability in comprehension gains rather than causal treatment effects. Because no randomized comparison group or counterfactual estimation framework was implemented, all predictive outputs were interpreted as associative and exploratory rather than causal.

##### Cross-Validation and Leakage-Prevention Procedures

To minimize information leakage and overfitting, all prediction-related analyses were conducted within a 10-fold cross-validation framework.

Within each training fold, preprocessing procedures, including z-score standardization and feature construction, were performed exclusively using training data. The resulting preprocessing parameters were subsequently applied to the corresponding validation fold.

Hyperparameter optimization was conducted independently within each training fold using Bayesian optimization for XGBoost and LightGBM and grid search for Random Forest and Elastic Net models. SHAP analyses were performed after model fitting within each training fold without using validation observations during model development.

Prediction performance was evaluated using R^2^, RMSE, MAE, and calibration error across all validation folds. This approach minimized information leakage and improved the robustness of model generalization.

#### 2.7.4. Nonlinear Cognitive Landscape Modeling

Three-dimensional nonlinear response surfaces and predicted gain landscapes were derived to assess multidimensional relationships among regulation, evaluation, metacognitive knowledge, readiness states, and predicted comprehension gains. Multidimensional cognitive space was analyzed using contour topology and marginal-effect analyses to identify adaptive zones, lower-response regions, and transitional regions.

#### 2.7.5. Exploratory Markov Learner-State Redistribution Analysis

An exploratory discrete-time Markov analysis was performed to examine learner-state redistribution patterns between pre-intervention and post-intervention observations. Because only two observation periods were available, the analysis was intended to provide descriptive information regarding learner-state redistribution rather than characterize long-term response distribution. Transition matrices, persistence probabilities, entropy measures, and mobility indices were estimated to summarize learner-state changes between observation periods.

To identify learner-state redistribution patterns, transitional states and persistent learner categories, the learner-state structure was analyzed using the tools for transition entropy and persistence. Additional learner-state redistribution and exploratory simulation diagnostics are shown in Supplementary Figure S4.

Learner states were determined at two empirical observation periods: baseline (pre-intervention) and post-intervention. Participants were assigned to one of four learner phenotypes (High Responders, Strategic Improvers, Monitoring-Dependent Learners, and Low Responders) based on the phenotype classification framework. Transition probabilities were estimated by comparing phenotype membership between these two points.

The transition matrix was constructed by calculating the relative frequencies of movement from each baseline state to each post-intervention state. State persistence was defined as the proportion of participants remaining in the same state across both observations, whereas transition entropy was calculated to quantify the degree of uncertainty in state redistribution. Because only two observation periods were available, the Markov analyses (descriptive learner-state redistribution) were interpreted descriptively and should not be considered evidence of long-term developmental trajectories or stable transitional region.

#### 2.7.6. Bayesian Adaptive Modeling and Reinforcement-Learning Optimization

Bayesian adaptive inference and reinforcement-learning optimization were performed exclusively after completion of the intervention and therefore did not influence classroom instruction. These procedures were implemented as exploratory computational simulations to investigate hypothetical adaptive instructional scenarios under varying learner-state conditions.

The individualized optimization of intervention and adaptation of the learner state were modeled using Bayesian adaptive inference and reinforcement-learning architectures in the ACIA framework. Reinforcement-learning optimization was not implemented during the classroom intervention and did not guide instructional decisions for participating students. Instead, exploratory simulation was conducted offline after data collection as an exploratory simulation-based procedure to investigate hypothetical adaptive instructional scenarios under varying learner-state conditions. Bayesian posterior estimation incorporated multidimensional metacognitive and adaptive-intervention parameters.

Four parallel Markov Chain Monte Carlo (MCMC) chains, each consisting of 5000 iterations and 1000 warm-up iterations, were used for estimation. The R-hat diagnostics and the estimation of effective sample size were used to assess convergence stability. Complete posterior estimates and convergence statistics are given in Supplementary Table S7.

Modeled adaptive reward trajectories, intervention-intensity calibration, and individualized instructional-policy assignment across learner states using reinforcement-learning optimization. Additional reinforcement-learning simulation diagnostics and reward-transition topologies are provided in Supplementary Figure S5.

In the reinforcement-learning framework, latent cognitive-response phenotypes from the clustering analysis were used to represent learner states, while intervention actions were used to refer to various adaptive instructional strategies such as regulation training, monitoring support, evaluative scaffolding, and foundational intervention support. The adaptive reward function was defined as expected comprehension gain, modified by a cognitive-risk penalty, thus emphasizing instructional policies that are known to promote both greater learning and less cognitive instability.

We adopted Q-learning with a greedy exploration strategy to optimize the policy in the iterative learning step, while simultaneously drawing interventions from a variety of policy levers, a strategy that has proven to be effective when combined with policy intervention assignment. The reinforcement-learning environment consisted of four learner states corresponding to the identified learner phenotypes (High Responders, Strategic Improvers, Monitoring-Dependent Learners, and Low Responders). The action space consisted of four hypothetical instructional actions: regulation training, monitoring support, evaluative scaffolding, and foundational support. The adaptive reward function was defined as predicted comprehension gain adjusted by a cognitive-risk penalty to prioritize instructional policies that simultaneously maximize learning gains and minimize learner vulnerability. An ε-greedy exploration strategy was implemented using an initial exploration rate (ε) of 0.20, which decayed by a factor of 0.995 after each episode until reaching a minimum value of 0.01. The learning rate (*α*) was set at 0.10 and the discount factor (*γ*) at 0.95. The reinforcement-learning simulation was performed for 1000 episodes to estimate stable instructional-policy trajectories. The updated process of the Q-value was then followed:

$$Q(s,a)\leftarrow Q(s,a)+\alpha [r+\gamma maxQ(s^{\prime },a^{\prime })-Q(s,a)]$$

The learner state *Q* (*s*, *a*) is the expected reward that comes with the learner state *s* and the instructional action a, *α* denotes the learning rate, *r* the adaptive reward and *γ* the future reward-discount factor. This approach allowed flexible optimization of personalized learning paths through multiple states of learner response.

Bayesian hierarchical modeling was specified as:

$$y_{i}∼\mathrm{Normal}(\mu_{i},\sigma )$$

where $y_{i}$ represents learner responsiveness (predicted comprehension gain) for participant i.

The linear predictor was defined as:

$$\mu_{i}=\beta_{0}+\beta_{1}({\mathrm{Planning}}_{i})+\beta_{2}({\mathrm{Monitoring}}_{i})+\beta_{3}({\mathrm{Evaluation}}_{i})\;\;\;\;\;+\beta_{4}({\mathrm{Metacognitive}\;\mathrm{Knowledge}}_{i})+\beta_{5}({\mathrm{Strategy}\;\mathrm{Flexibility}}_{i})$$

Weakly informative priors were assigned as follows:

$$\beta_{j}∼\mathrm{Normal}(0,5)$$

for all regression coefficients $\left(j=0,\ldots ,5\right)$, and

σ∼HalfNormal(5)

for the residual standard deviation parameter.

Posterior estimation was performed using four parallel MCMC chains, each consisting of 5000 iterations with 1000 warm-up iterations. Convergence was evaluated using the potential scale reduction factor (R-hat < 1.01), effective sample size diagnostics, and visual inspection of posterior trace plots. Complete posterior estimates and convergence diagnostics are provided in Supplementary Table S7.

##### Reinforcement-Learning Simulation Specifications

Reinforcement learning was performed exclusively as an exploratory post hoc simulation after completion of the intervention and was not implemented with real learners during classroom instruction. A Q-learning framework was used to investigate hypothetical adaptive instructional scenarios under different learner-state conditions. Learner states corresponded to the four identified learner phenotypes, whereas instructional actions represented pedagogically plausible adaptations, including increasing monitoring support, evaluative reflection, strategic-flexibility exercises, or maintaining instructional intensity. State-transition probabilities were estimated from the observed learner-state redistribution patterns. The reward function was designed to maximize predicted comprehension gains while penalizing excessive instructional complexity (Table 2). All reinforcement-learning hyperparameters used in the simulation, including the learning rate, discount factor, exploration schedule, and stopping criteria, are summarized in Table 2 to facilitate reproducibility.

#### 2.7.7. Robustness, Uncertainty, and Overfitting Assessment

Although the analytical pipeline incorporated multiple computational components, several safeguards were implemented to reduce overfitting risk and improve model robustness. All preprocessing procedures, feature standardization, hyperparameter optimization, and interpretability analyses were performed exclusively within training folds before application to validation folds to prevent information leakage.

Because the sample size was modest (n = 180) relative to the complexity of the analytical framework, additional robustness checks were conducted. Model uncertainty was quantified using bootstrapped confidence intervals for predictive performance metrics and SHAP-based predictor importance values. Training–validation performance gaps were also examined to evaluate possible overfitting.

The stability of the learner-response phenotype solution was assessed using silhouette coefficients, Davies–Bouldin indices, Bayesian Information Criterion values, and profile-membership agreement across repeated resampling procedures. Bayesian uncertainty was evaluated through posterior credible intervals, effective sample sizes, and convergence diagnostics.

Despite these validation procedures, the findings should be interpreted cautiously because the analytical framework was developed using a single-institution sample. External validation using independent institutional datasets is required before claims regarding scalability, deployment, or generalizability can be made. Additional robustness diagnostics are provided in Supplementary Table S5 and Supplementary Figure S6.

### 2.8. Exploratory Computational Benchmarking

Because the study did not include a randomized control group or a non-adaptive instructional comparison group, comparative analyses were not intended to establish instructional superiority or causal effectiveness. Instead, exploratory computational benchmarks were constructed to contextualize possible adaptive instructional scenarios under alternative assumptions.

Three hypothetical reference conditions were generated: (i) a uniform metacognitive instruction condition, (ii) a single-component instructional condition, and (iii) a historical baseline reference estimated from pre-intervention learner performance. These benchmarks were used exclusively as simulation-based reference scenarios for evaluating predicted comprehension gains, learner-state transitions, and adaptive reward estimates.

These analyses should not be interpreted as empirical comparisons because all participants received the same classroom-based intervention and no randomized assignment was performed. Consequently, the benchmarking framework is presented only as a hypothesis-generating exercise for future randomized controlled studies.

### 2.9. Computational Environment and Reproducibility

All statistical analysis, explainable machine-learning procedures, Bayesian adaptive inference, and reinforcement-learning simulations were performed in Python v3.11. Computational modeling included using the following libraries: Scikit-learn, XGBoost, LightGBM, SHAP, PyMC, NumPy, Pandas, Matplotlib (version 3.10.0), Seaborn (version 0.13.2), and NetworkX (version 3.4.2) in a reproducible analytical environment. Random seeds were fixed across all modeling procedures to ensure reproducibility. The random seed was fixed at 42 across all machine-learning procedures to ensure reproducibility of clustering, cross-validation, hyperparameter optimization, and predictive modeling.

### 2.10. Supplementary Material

Validation analyses for the participant-selection workflow, latent learner-response phenotyping, SHAP interaction modeling, learner-state redistribution, reinforcement-learning simulations, Bayesian posterior diagnostics, sensitivity analyses, and robustness evaluations are provided in Supplementary Tables S1–S7 and Supplementary Figures S1–S6.

## 3. Results

A significant heterogeneity was observed in students’ responses to the metacognitive intervention, resulting in the identification of four learner-response phenotypes. Unless otherwise specified, all computational analyses reported in this section were conducted after completion of the intervention and should be interpreted as post hoc analytical procedures rather than components of the classroom instructional implementation.

**Figure 1:**
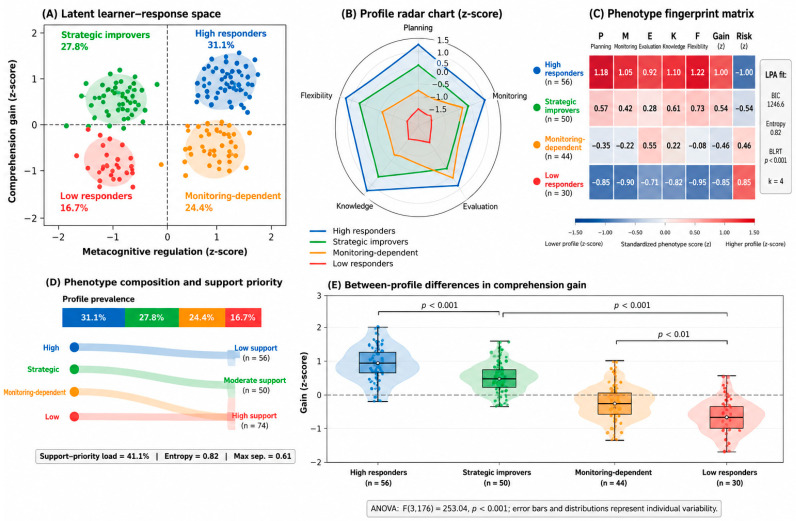
Baseline-derived learner-response phenotypes and multidimensional metacognitive fingerprints. Learner-response phenotypes were identified exclusively using baseline metacognitive variables (planning, monitoring, evaluation, metacognitive knowledge, and strategy flexibility). Reading-comprehension gain is presented only as an external post-intervention outcome variable to characterize the identified phenotypes and was not used during phenotype construction.

High Responders occupied the upper-right region of the latent learner-response space and exhibited high levels of metacognitive regulation together with substantial improvements in reading comprehension. Strategic Improvers demonstrated moderate-to-high levels of strategy flexibility and positive improvement patterns, indicating developing but not fully consolidated self-regulatory structures (Figure 1). Monitoring-Dependent Learners exhibited relatively preserved monitoring abilities but lower levels of evaluation and information integration, whereas Low Responders were clustered in the lower-left region and displayed lower levels of regulatory functioning and consistently lower comprehension performance. The spatial separation between phenotypes indicates heterogeneous learner-response patterns rather than a uniform intervention effect. The complete participant demographic, academic, and metacognitive-background characteristics are presented in Supplementary Table S1.

The multidimensional metacognitive fingerprint analysis revealed clear differences among learner-response phenotypes in planning, monitoring, evaluation, metacognitive knowledge, and strategy flexibility. High Responders demonstrated the strongest integrated regulatory organization and the lowest cognitive-risk burden, whereas Low Responders exhibited consistently negative standardized scores and greater regulatory vulnerability. Strategic Improvers demonstrate moderate positive scores across most dimensions, suggesting substantial developmental potential, whereas Monitoring-Dependent Learners exhibited fragmented self-regulatory organizations characterized by relatively preserved monitoring abilities but limited evaluative control. The phenotype composition analysis further indicated that approximately 41% of students belonged to higher-support categories, suggesting that future individualized instructional approaches may be beneficial for specific learner groups.

Statistical comparisons of reading-comprehension gains confirmed significant differences among learner-response phenotypes. High Responders exhibited the largest and most consistent comprehension improvements, whereas Strategic Improvers demonstrated moderate but more variable improvements. Monitoring-Dependent Learners showed near-neutral gain distributions, while Low Responders consistently exhibited the lowest gain patterns. ANOVA and post hoc comparisons indicated significant differences in learner responsiveness across phenotypes, supporting the presence of heterogeneous learner responses rather than uniform patterns of change. These findings suggest that learners may benefit from future individualized instructional approaches; however, the present study was not designed to establish the superiority of precision-oriented educational interventions or causal instructional effectiveness.

Descriptive and psychometric analyses showed that the distribution of all metacognitive dimensions was acceptable and demonstrated high internal consistency. The mean scores for planning, monitoring, evaluation, metacognitive knowledge, and strategy flexibility were all moderate to high among the participants. Skewness and kurtosis values were within acceptable normality ranges, which supported the application of subsequent multivariate and nonlinear analytical procedures. Reliability analysis showed high internal consistency across the metacognitive constructs (ranging from Cronbach’s *α* = 0.83 to McDonald’s ω = 0.89), thus supporting construct stability for latent learner-response phenotyping, explainable machine-learning modeling, and post hoc computational analyses (Table 3).

Learner-response phenotypes were identified exclusively from baseline metacognitive variables using Gaussian Mixture Modeling. Reading-comprehension gain was intentionally excluded from the clustering procedure and was analyzed only after phenotype identification as an external post-intervention outcome variable to characterize differences among the identified learner groups. There was significant heterogeneity in learner responses to the metacognitive intervention, as revealed by the latent phenotype characterization. The largest subgroup consisted of High Responders (31.1%; n = 56), followed by Strategic Improvers (27.8%; n = 50), Monitoring-Dependent Learners (24.4%; n = 44), and Low Responders (16.7%; n = 30) (Table 4). High Responders exhibited the highest standardized scores across planning, monitoring, evaluation, and strategy flexibility, together with the greatest external post-intervention reading-comprehension gains and the lowest cognitive-risk index. Strategic Improvers demonstrated moderately positive metacognitive profiles and intermediate comprehension gains. Monitoring-Dependent Learners showed relatively preserved monitoring ability but comparatively lower planning, evaluation, and strategy flexibility. Low Responders consistently exhibited negative standardized metacognitive scores, the lowest external comprehension gains, and the highest cognitive-risk index. These findings support substantial heterogeneity in learner-response patterns following the intervention.

**Figure 2:**
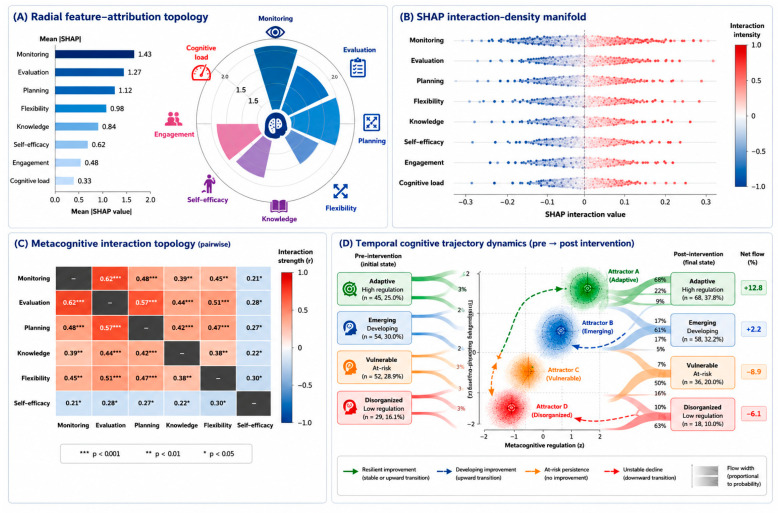
Explainable machine-learning architecture showing nonlinear metacognitive determinants, interaction topology, and adaptive predicted gain landscapes.

Post hoc explainable machine-learning analyses identified that monitoring, evaluation, and planning were the most salient metacognitive factors associated with heterogeneous improvements in reading comprehension. Results of the radial attribution topology indicated that higher-order regulatory processes contributed more strongly to prediction performance than motivational or contextual variables, suggesting that comprehension gains were associated with an integrated self-regulatory architecture rather than with isolated cognitive traits (Figure 2). The SHAP interaction-density manifold also showed strong interactions among the monitoring, strategy flexibility, and metacognitive knowledge dimensions, indicating that the effect of one regulatory process was dependent on the levels of complementary metacognitive processes. The extended SHAP dependence diagnostics and the nonlinear interaction topologies are shown in Supplementary Figure S3. Positive SHAP interaction regions were localized in high-intensity regulatory profiles, while fragmented or lower-intensity profiles had lower contributions to predictions and were associated with lower comprehension gains.

A metacognitive interaction topology was applied and revealed strong interactions among monitoring, evaluation, planning, and metacognitive knowledge dimensions, showing that these functioned as an integrated regulatory network rather than autonomous cognitive variables. The highest interaction strengths were found for monitoring–evaluation and evaluation–planning interactions. The multidimensional learner-response landscape indicated regions associated with higher and lower comprehension gains. High-gain regions coincide with high levels of planning and integrated evaluative planning, while low-gain regions coincide with more fragmented monitoring structures and less coherent planning. Moderate strategic organization in transitional profiles showed intermediate improvement potential, indicating that learners exhibited gradual variation in their responses to the intervention rather than representing entirely separate learner categories.

The predictive-validation analysis showed satisfactory predictive performance of the post hoc explainable machine-learning models within the study dataset. The highest prediction accuracy (R^2^ = 0.61; RMSE = 0.58) was observed for the XGBoost model, with LightGBM and Random Forest models performing similarly, while Elastic Net performed comparatively poorly (Table 5). The cross-validation results demonstrated stable generalization performance, with low variability in prediction accuracy across folds. Calibration errors across the ensemble-based approaches were low, and there was good agreement between predicted and observed comprehension gains. Together, these results indicate that the estimated learner-response patterns can predict heterogeneous metacognitive response patterns within this dataset. These findings should be interpreted as predictive performance estimates rather than evidence of instructional effectiveness, causal inference, or real-time adaptive instructional implementation.

The configuration and optimization details of the explainable machine-learning models are presented in Supplementary Table S2.

**Figure 3:**
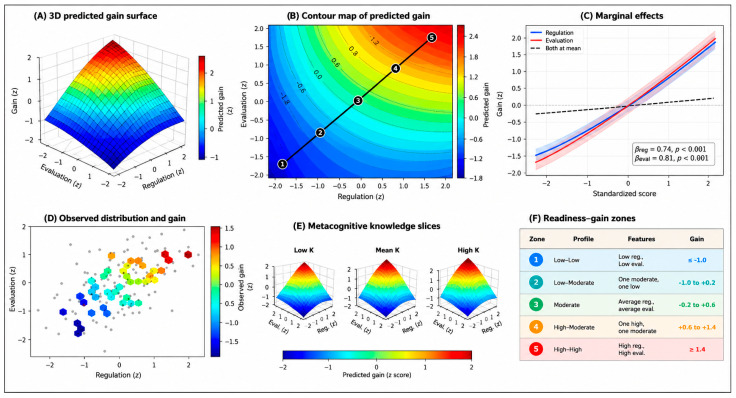
Nonlinear cognitive readiness–gain landscape showing predicted comprehension improvement as a function of baseline metacognitive Planning, evaluation, and metacognitive knowledge levels (N = 180). Eval. Evaluation, Reg. Regulation.

Predicted comprehension gains increased with higher baseline metacognitive planning and evaluation, as shown in the nonlinear response surface and contour topology (Figure 3A,B). The readiness continuum, extending from low to high readiness, demonstrated a steady increase in predicted gains, suggesting that simultaneous improvements in planning and evaluative monitoring may have synergistic associations with reading-comprehension performance. Marginal-effect analysis also revealed that evaluation had a slightly larger positive effect than planning, although both dimensions exhibited significant positive associations with comprehension gains along the standardized cognitive continuum.

Observed student distributions revealed clear clusters of students with higher readiness scores in regions associated with positive comprehension gains, whereas students with lower readiness scores were concentrated in regions associated with lower comprehension gains. The metacognitive knowledge slices revealed that higher levels of metacognitive knowledge shifted the entire predictive surface upward, suggesting that declarative and procedural metacognitive knowledge may enhance the contribution of planning and evaluative control during comprehension tasks.

The readiness–gain classification model revealed five interpretable readiness–gain zones, ranging from low-readiness/low-gain profiles to high-readiness/high-gain profiles. Students located in higher-readiness regions demonstrated the highest predicted gains, whereas students located in lower-readiness regions demonstrated substantially lower predicted gains. Together, this multidimensional space suggests nonlinear associations between metacognitive readiness and comprehension outcomes, whereby relatively small changes in metacognitive readiness may be associated with larger differences in comprehension performance.

The exploratory Markov analysis revealed non-uniform learner-state redistribution patterns between pre-intervention and post-intervention observations. High Responders demonstrated the highest persistence probability, whereas Strategic Improvers showed moderate mobility toward higher-support learner profiles. Monitoring-Dependent Learners exhibited intermediate redistribution patterns, while Low Responders remained comparatively stable over time. Because only two observation periods were available, these findings should be interpreted as descriptive learner-state redistribution patterns rather than evidence of long-term response distribution.

**Figure 4:**
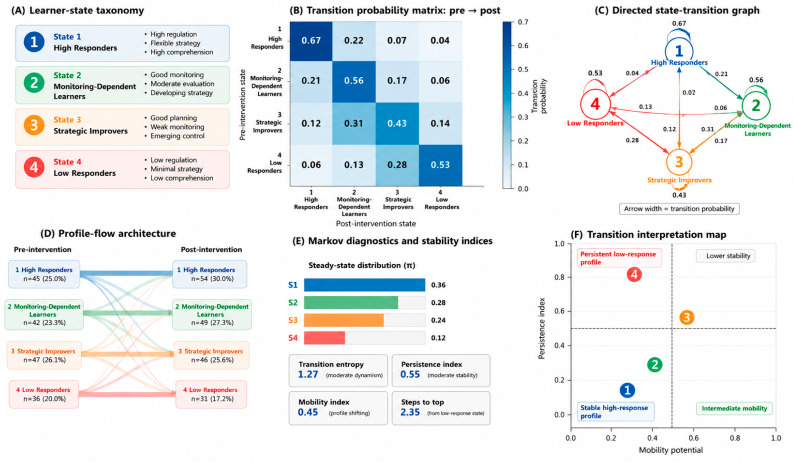
Markov transition architecture of learner-profile dynamics across metacognitive intervention.

Strategic Improvers also displayed moderate persistence (0.56), whereas Monitoring-Dependent Learners demonstrated greater mobility and transitioned toward Strategic Improvers and High Responders profiles. Low Responders exhibited a relatively high persistence probability (0.53), indicating that lower regulatory functioning may be less susceptible to rapid change and may require longer intervention periods or additional instructional support (Figure 4). The transition matrix and directed state-transition graph showed asymmetric learner-state redistribution patterns rather than random redistribution across profiles. Redistribution patterns were primarily directed toward intermediate learner profiles, with the largest number of transitions occurring between Monitoring-Dependent Learners and Strategic Improvers (0.31). In contrast, there was substantially less direct movement from low-response profiles to high-response profiles, suggesting that improvements in metacognitive functioning may occur gradually rather than abruptly.

The profile-flow architecture descriptively illustrated learner-state redistribution following the intervention. However, because only two observation periods were available and no comparison group was included, these findings should not be interpreted as evidence of causal developmental progression. Transition entropy (H = 1.27) indicated organized variability in learner-state redistribution, whereas the persistence index (0.55) indicated moderate stability between observation periods. According to the transition interpretation map, State 1 demonstrated the highest persistence probability, whereas State 4 exhibited the greatest persistence among low-response profiles. State 3 occupied an intermediate position with relatively high mobility potential, suggesting that this profile may represent an important intermediate stage in learner-state redistribution where additional instructional support may be beneficial.

The transition probability matrix showed that learner-state redistribution patterns were asymmetric and stage dependent following the metacognitive intervention. High Responders showed the highest persistence probability (0.67), indicating high stability of advanced self-regulated learning patterns over time (Table 6). Strategic Improvers demonstrated moderate persistence (0.56), whereas Monitoring-Dependent Learners displayed the highest mobility, particularly toward Strategic Improvers and High Responders profiles. Low Responders maintained a relatively high self-transition probability (0.53), indicating that lower regulatory functioning may remain relatively stable and may be less likely to transition rapidly toward higher-performing profiles. Direct transitions from low-response profiles to high-response profiles were comparatively infrequent, suggesting that improvements in metacognitive functioning may occur gradually rather than through abrupt changes. These findings support the presence of nonlinear learner-state redistribution patterns and heterogeneous learner responses following the intervention. The latent-profile stability and predictive calibration results are provided in Supplementary Figure S2.

**Figure 5:**
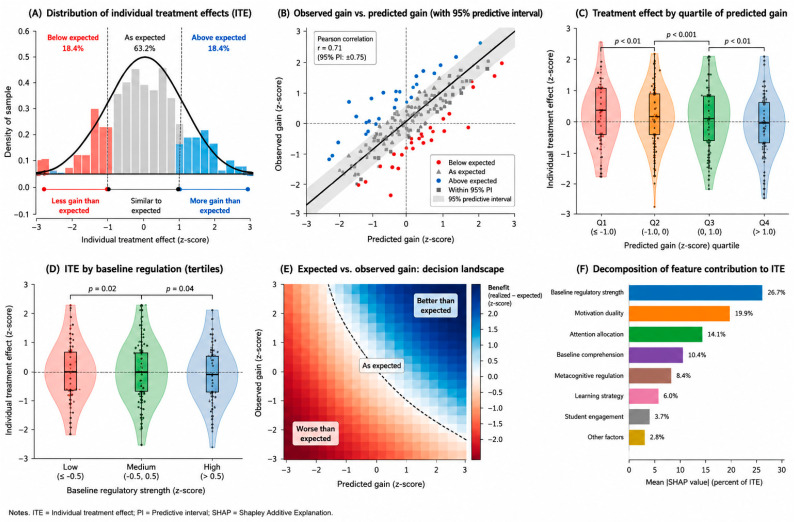
Individual learner-response heterogeneity analysis illustrating distributions of learner-specific intervention gains, predictive accuracy, subgroup variability, nonlinear deviation topology.

The learner-response heterogeneity analysis revealed significant variation in learners’ responses to the metacognitive intervention. Individual intervention-associated gain distributions revealed that 28.3% of students achieved greater gains than those predicted by the model, whereas another 28.3% achieved gains lower than predicted, suggesting substantial inter-individual variability in learner responses. Most students (43.3%) were within the expected improvement range, indicating acceptable overall model calibration. The observed versus expected comparison also demonstrated satisfactory predictive performance (R^2^ = 0.56; RMSE = 0.64); however, there remained residual learner heterogeneity that was not fully captured by conventional linear assumptions, as several learners performed substantially better than predicted at baseline, whereas others performed substantially worse (Figure 5).

Quartile-based analyses indicated systematic variation in learner responses according to predicted learner profiles and baseline metacognitive planning. Likewise, significant differences in positive deviations from expected improvement were observed between high- and low-planning learners (*p* < .05). These findings suggest that metacognitive readiness may influence learners’ responses to the intervention and subsequent comprehension gains. The deviation heatmap and SHAP decomposition analysis further revealed that planning-related metacognitive processes were among the most influential factors associated with learner responses at the individual level. Positive deviations from expected gains were concentrated in regions characterized by higher predicted gains and stronger metacognitive planning, whereas negative deviations were concentrated in lower-readiness regions. Additionally, SHAP decomposition showed that baseline metacognitive planning (26.7%), evaluation (19.8%), and planning ability (16.1%) contributed most strongly to intervention-associated gains, whereas academic engagement and auxiliary factors contributed comparatively less. Phenotype-level inferential statistics and additional post hoc comparisons are presented in Supplementary Table S6.

**Figure 6:**
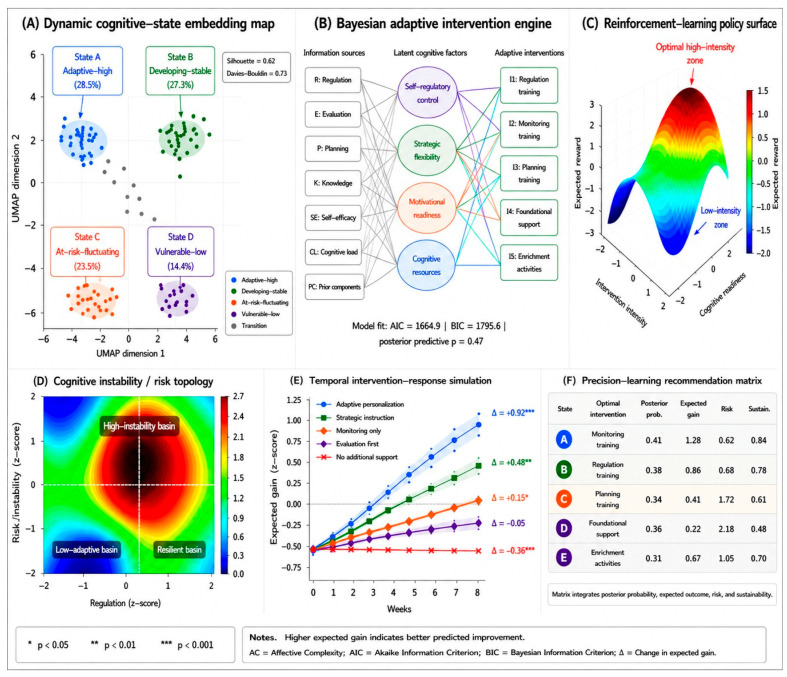
Adaptive Cognitive Intervention Architecture illustrates latent cognitive state.

The ACIA uncovered unique learner-response phenotypes distributed across a nonlinear learner-response space. Four learner-response phenotypes were identified: High Responders, Strategic Improvers, Monitoring-Dependent Learners, and Low Responders. The clustering topology showed significant separation between learner-response phenotypes (silhouette = 0.62; Davies–Bouldin = 0.71), indicating stable latent differentiation among participants. High Responders demonstrated strong self-regulatory organization, particularly in planning and strategic monitoring, whereas Low Responders exhibited weaker self-regulatory organization, including lower planning and strategic-monitoring abilities. Monitoring-Dependent Learners occupied intermediate regions between higher- and lower-response profiles, suggesting heterogeneous learner-response patterns following the intervention rather than stable long-term learner-state evolution (Figure 6).

The Bayesian adaptive inference framework and reinforcement-learning simulation surface showed that hypothetical adaptive instructional scenarios were highly dependent on learner-response profiles and metacognitive readiness. Bayesian posterior estimation suggested that planning and monitoring support strategies were more strongly associated with higher-response learners, whereas foundational support mechanisms were more frequently associated with lower-response learners. The reinforcement-learning simulation surface also revealed a nonlinear reward topology, whereby moderate-to-high instructional intensity was associated with higher expected gains when metacognitive readiness was high. However, lower-readiness learners exposed to high instructional intensity were associated with overload-risk regions and lower expected rewards, suggesting that instructional intensity may need to vary according to learner characteristics.

The ACIA exploratory computational framework produced the largest simulated improvement pattern over the eight-week intervention period (Δ +0.92); however, these findings represent simulation-based scenarios rather than empirical comparisons with alternative instructional approaches. Supplementary Figures S4 and S5 and Supplementary Table S7 provide additional details regarding exploratory simulation analyses, Bayesian posterior estimates, and reinforcement-learning simulation diagnostics. Collectively, these findings support ACIA as an exploratory computational framework in which learner responses may be heterogeneous, probabilistic, and nonlinear, rather than assuming a single uniform intervention pattern for all learners.

## 4. Discussion

The results should be interpreted within the limitations of a single-group quasi-experimental design and a post hoc computational framework rather than as evidence supporting a fully deployed real-time adaptive educational system. The present study explored heterogeneous learner-response patterns during a metacognitive reading intervention within an exploratory computational framework. The findings revealed significant inter-individual differences in comprehension gains, highlighting the importance of metacognitive planning and individualized instructional support in higher education contexts.

It is important to emphasize that the classroom intervention and the computational analyses represented two distinct components of the study. The eight-week metacognitive intervention was empirically implemented in classroom settings, whereas learner-response phenotyping, predictive modeling, Bayesian adaptive inference, and reinforcement-learning procedures were subsequently conducted as post hoc analytical and simulation-based procedures after completion of data collection.

Before interpreting the findings, it is necessary to distinguish between empirical observations, predictive modeling outputs, and simulation-based analyses. The observed pre–post changes were limited to outcomes associated with the eight-week classroom-based metacognitive intervention. Explainable machine-learning and Bayesian approaches were used to estimate heterogeneous learner-response patterns and predictive relationships, whereas reinforcement-learning procedures were conducted exclusively as post hoc simulations to explore hypothetical adaptive instructional scenarios. Consequently, the findings should not be interpreted as evidence of causal superiority but rather as an exploratory computational framework that may inform future randomized and real-time adaptive educational studies.

Importantly, all participants received the same metacognitive instructional program, and ACIA was not deployed as an operational adaptive instructional system. Instead, ACIA functioned as a post hoc computational framework for analyzing learner heterogeneity and exploring simulation-based instructional scenarios. Therefore, the findings should be interpreted as exploratory evidence supporting computational feasibility rather than validation of an operational adaptive intervention platform.

Furthermore, the findings should not be interpreted as evidence that ACIA outperforms traditional instructional approaches because the study did not include a randomized control group or a non-adaptive comparison condition. Rather, the study provides evidence regarding learner heterogeneity, predictive-modeling performance, and exploratory simulation-based analyses within the proposed computational framework.

### 4.1. Emergence of Learner-Response Phenotypes and Cognitive Heterogeneity

The study showed that the metacognitive intervention was associated with considerable heterogeneity in learners’ responses, resulting in four distinct learner-response phenotypes characterized by different regulatory structures and comprehension-gain patterns. High Responders demonstrated stronger planning, strategy flexibility, and evaluative control, whereas Low Responders exhibited weaker and less consistent gains together with fragmented self-regulatory organization (Bürgler et al., 2021). The results suggest that metacognitive instructional outcomes may not arise solely from standardized intervention exposure but may vary according to heterogeneous learner-response patterns and individualized instructional support needs. These findings are consistent with the metacognitive frameworks proposed by Flavell (1979) and Zimmerman (2002), which emphasize the importance of monitoring, regulation, and evaluative control in self-regulated learning. Likewise, Azevedo and Cromley (2004) reported that learners’ self-planning positively influences reading comprehension during technology-supported learning. The present study adopted a person-centered computational modeling approach that identified latent learner-response phenotypes, thereby supporting a learner-centered interpretation of metacognitive development compared with traditional variable-centered approaches.

The pedagogical approach used during the intervention may have contributed to the observed variation in learner responses. Explicit planning prompts, monitoring checklists, reflective journals, guided questioning, and formative feedback mechanisms may have facilitated the gradual internalization of self-regulatory reading strategies. The staged instructional design also enabled learners with different readiness levels to engage in metacognitive planning with varying degrees of instructional support, which is consistent with contemporary self-regulated learning theories that emphasize scaffolded autonomy and individualized instructional support.

### 4.2. Metacognitive Coordination and Predictive Learning Patterns

An analysis of the explainable machine-learning models showed that monitoring, evaluation, planning, and strategy flexibility were the most influential nonlinear predictors of comprehension gains across learners. In addition, the SHAP interaction topology further validated that these metacognitive dimensions functioned as interconnected processes rather than separate entities. Regions associated with positive comprehension gains were identified in coordinated planning and evaluative-control structures, whereas lower-gain regions were associated with fragmented monitoring structures (Matta, 2026). These findings are consistent with the theoretical perspectives of Schraw and Dennison (1994) and Greene and Azevedo (2007), who proposed that self-regulated learning results from coordinated interactions among planning, monitoring, and evaluative processes. The superior performance of XGBoost and LightGBM compared with Elastic Net also suggests that metacognitive learning systems operate through nonlinear relationships that may not be adequately captured by traditional linear models.

The results are also consistent with theoretical perspectives derived from Cognitive Load Theory (Kalyuga, 2023). Learners with stronger regulatory coordination may be better able to manage cognitive demands and adjust comprehension strategies when reading tasks become more complex.

### 4.3. Transitions in Readiness and Growth

The findings from the nonlinear readiness–gain landscape and Markov analyses suggest that learner changes occurred gradually rather than through abrupt transitions. Learners with higher readiness scores were associated with regions characterized by stronger planning and metacognitive knowledge, whereas learners with lower readiness scores were associated with lower improvement potential and fragmented regulatory organization.

The transition entropy and persistence indices provide evidence supporting learner-state redistribution patterns during the intervention period (Luckin et al., 2016; Hecht & Crowley, 2020). High Responders exhibited the highest probability persistence, whereas Low Responders demonstrated greater resistance to change (Li et al., 2026). However, because only two observation periods were available, these findings should be interpreted as exploratory learner-state redistribution patterns rather than evidence of long-term response distribution. Collectively, the results suggest that learners with different levels of metacognitive readiness may require different levels of instructional support.

### 4.4. Learner-Response Heterogeneity and Metacognitive Readiness

The heterogeneity analysis of intervention-associated gains revealed significant between-individual differences in learner responses to the metacognitive intervention. Learners who initially exhibited higher levels of planning and evaluative readiness also demonstrated significantly greater positive deviations from expected improvement, suggesting that metacognitive readiness may act as an important moderating factor influencing intervention-associated improvements (Fidan & Koçak Usluel, 2024).

These findings are consistent with previous studies showing that self-regulatory readiness and strategic learning abilities significantly influence learners’ responses to instructional interventions (Dignath & Büttner, 2008; Donker et al., 2014; Chen, 2022). However, the present study extends previous work by integrating SHAP decomposition, entropy-based analyses, and intervention-associated gain analyses within an exploratory computational framework that allows the modeling of nonlinear individual contribution patterns.

### 4.5. ACIA as an Exploratory Computational Framework for Individualized Instructional Support

The ACIA suggests that metacognitive learning can be conceptualized as a heterogeneous and probabilistic learning process rather than a dynamically adaptive teaching-learning system. Bayesian adaptive inference and reinforcement-learning simulations indicated that hypothetical adaptive instructional scenarios may vary according to learner-response profiles and metacognitive readiness (Bauer & Gharabaghi, 2015). However, these findings represent post hoc simulation-based analyses and should not be interpreted as empirical evidence that personalized instructional approaches outperform traditional instructional strategies.

These findings are consistent with recent studies in educational artificial intelligence suggesting that adaptive learning systems and learning analytics may support individual learning when combined with learner modeling and probabilistic decision frameworks (Baker & Inventado, 2014; Luckin et al., 2016). The present study extends this literature by integrating latent learner-response phenotyping, explainable machine learning, Bayesian adaptive inference, Markov transition analysis, and reinforcement-learning simulations within a single exploratory computational framework.

The proposed framework may also have future applications in higher education environments through integration with Learning Management Systems and learning analytics platforms (Sajja et al., 2024). In future implementations, ACIA could support the development of instructional dashboards capable of identifying learners who may require additional support, recommending metacognitive strategies, and monitoring reading-comprehension development over time. However, such applications remain hypothetical and were not implemented or empirically validated in the present study. Therefore, these findings should be interpreted as exploratory possibilities for future research rather than evidence of a real-time adaptive instructional system.

### 4.6. Practical Implications for Individualized Instructional Support in Higher Education

The results of the present study have important implications for the future development of individualized instructional support and metacognitive learning environments in higher education. The proposed ACIA framework may facilitate the future development of learning platforms integrated with Learning Management Systems that can help identify lower-response learner profiles, monitor self-regulatory difficulties, and recommend metacognitive support strategies according to learner characteristics (Liu, 2022).

The explainable machine-learning architecture may also help instructors identify dominant regulatory deficits associated with lower comprehension gains and facilitate pedagogical scaffolding and early academic support. Likewise, the combination of Bayesian adaptive inference and reinforcement-learning simulations may support the future development of intelligent tutoring systems capable of adjusting instructional intensity according to learner-response patterns (Halkiopoulos & Gkintoni, 2024). At the institutional level, the framework may support educational analytics dashboards designed to facilitate academic advising, reading-support programs, and self-regulated learning strategies.

In this way, ACIA provides a potential computational foundation for future higher education applications that may incorporate explainable artificial intelligence, instructional support systems, and learner-monitoring approaches. However, these applications remain conceptual and were not implemented or empirically validated in the present study.

### 4.7. Limitations

Several limitations should be considered when interpreting the findings. First, the study employed a single-group quasi-experimental design without a randomized control group, non-adaptive comparison condition, or a counterfactual estimation framework. Consequently, causal inference and comparative instructional effectiveness cannot be established. Second, the sample consisted of 180 undergraduate students from a single institutional setting. Although cross-validation, leakage-prevention procedures, uncertainty estimation, and robustness checks were implemented, the modest sample size relative to the complexity of the analytical pipeline may increase overfitting risk and limit the generalizability of the findings.

Third, external validation using independent institutional datasets was not performed. Therefore, conclusions regarding scalability, deployment, and broad applicability should be considered preliminary until validated in larger multi-institutional settings. Fourth, the intervention lasted only eight weeks, precluding evaluation of long-term metacognitive stabilization and sustained reading-comprehension development. Finally, the reinforcement-learning component was implemented exclusively as an offline post hoc simulation and was not deployed in a real-time adaptive educational environment. Therefore, its practical effectiveness remains hypothetical and requires empirical validation before implementation in authentic educational systems. Because no randomized comparison group or counterfactual estimation framework was implemented, all predictive analyses should be interpreted as exploratory assessments of learner-response variability rather than estimates of individualized treatment effects or causal relationships.

### 4.8. Future Directions

Future studies should employ randomized controlled, multi-institutional, and longitudinal designs to evaluate the effectiveness, stability, and generalizability of the proposed framework. Additional research should incorporate emotional, motivational, behavioral, and neurocognitive factors to develop more comprehensive learner-response models.

Future investigations may also evaluate real-time implementations of the framework within intelligent tutoring systems, learning-management systems, and educational analytics platforms. The integration of learner monitoring, intelligent dashboards, and reinforcement-learning simulations should be regarded as potential future applications that require independent empirical validation before educational deployment can be recommended.

### 4.9. Practical Implications

The findings provide preliminary evidence that computational approaches may help characterize learner heterogeneity during metacognitive reading interventions. The proposed framework may assist educators and researchers in identifying learner-response phenotypes and understanding variability in reading-comprehension development.

However, these implications should be interpreted cautiously. Real-time learner monitoring, learning-management-system integration, intelligent dashboards, and automated instructional assignments were not implemented in the present study and therefore should be regarded as potential future applications rather than validated educational technologies.

The exploratory computational framework may inform future data-informed instructional decision making and individualized learner support. Nevertheless, additional randomized, multi-institutional, and real-time implementation studies are required before practical deployment in authentic educational environments can be recommended.

## 5. Conclusions

The present study demonstrates the feasibility of integrating classroom-based metacognitive intervention data with computational modeling approaches to improve understanding of learner heterogeneity in higher education. The findings should be interpreted as exploratory evidence derived from an eight-week quasi-experimental intervention combined with post hoc computational analyses rather than as validation of a real-time adaptive instructional system.

The results suggest meaningful differences in metacognitive profiles, reading-comprehension gains, and learner-response patterns among participants. Planning, monitoring, evaluation, and strategy flexibility emerged as influential predictors of comprehension improvement, whereas learner-state redistribution analyses highlighted substantial heterogeneity in how students responded to the intervention.

The ACIA should be interpreted as an exploratory computational framework that integrates empirical intervention data, predictive modeling, and simulation-based analyses. Real-time learner monitoring, intelligent dashboards, learning-management-system integration, and reinforcement-learning deployment were not implemented and therefore remain potential future applications rather than validated outcomes.

Future research should employ larger, multi-institutional, randomized, and longitudinal designs together with independent external validation datasets to evaluate the scalability, generalizability, and practical applicability of the proposed framework before implementation in authentic educational environments can be recommended.

## Figures and Tables

**Table 1:** Computational workflow of the ACIA framework for precision-oriented metacognitive learning analysis.

Stage	Method	Input	Output	Purpose
Latent learner-response phenotyping	Gaussian Mixture Modeling (GMM) with expectation–maximization optimization	Baseline planning, monitoring, evaluation, metacognitive knowledge, and strategy flexibility	Four latent learner-response phenotypes	To identify heterogeneous baseline learner profiles while avoiding circular interpretation
Explainable machine-learning prediction	Random Forest, XGBoost, LightGBM, Elastic Net with 10-fold cross-validation	Baseline metacognitive variables, demographic characteristics, academic engagement indicators, and learner phenotype membership	Individualized comprehension-gain prediction	To model heterogeneous learner responsiveness
Developmental transition modeling	Discrete-time Markov transition analysis	Learner phenotypes estimated from pre- and post-intervention observations	Transition matrices, entropy, and persistence indices	To explore learner-state redistribution patterns between observation periods
Bayesian adaptive inference	Bayesian hierarchical modeling with MCMC estimation	Regulation, monitoring, evaluation, intervention intensity	Posterior estimates and credible intervals	To estimate probabilistic learner-response patterns and uncertainty structures through post hoc modeling
Reinforcement-learning optimization	Q-learning with ε-greedy exploration	Learner states, intervention actions, adaptive reward function	Optimized instructional-policy matrix	To simulate hypothetical adaptive instructional scenarios under different learner-state conditions

**Table 2:** Reinforcement-learning simulation specification.

Component	Specification
Algorithm	Q-learning
State space	High Responders, Strategic Improvers, Monitoring-Dependent Learners, Low Responders
Action space	Increase monitoring support; increase evaluative reflection; increase strategic flexibility; maintain instructional intensity
Reward function	Predicted comprehension gain − instructional complexity penalty
Risk penalty	Penalizes unstable and high-intensity instructional policies
Learning rate (*α*)	0.10
Discount factor (*γ*)	0.95
Exploration strategy	ε-greedy
Initial ε	0.20
Exploration decay	0.995
Minimum ε	0.01
Episodes	1000
Evaluation	Cumulative reward, convergence stability, policy consistency
Implementation	Offline post hoc simulation only
Real-time deployment	No

**Table 3:** Descriptive statistics and psychometric reliability of metacognitive and comprehension variables.

Variable	Mean	SD	Skewness	Kurtosis	Cronbach’s *α*	McDonald’s ω
Planning	3.71	0.64	−0.42	−0.31	0.86	0.87
Monitoring	3.84	0.59	−0.38	−0.27	0.88	0.89
Evaluation	3.67	0.66	−0.29	−0.41	0.85	0.86
Metacognitive Knowledge	3.79	0.61	−0.35	−0.22	0.84	0.85
Strategy Flexibility	3.58	0.71	−0.21	−0.47	0.83	0.84
Reading Comprehension Gain	0.74	0.69	0.18	−0.53	—	—

**Table 4:** Characteristics of baseline-derived learner-response phenotypes and external post-intervention reading-comprehension outcomes.

Phenotype	n	%	Planning (z)	Monitoring (z)	Evaluation (z)	Strategy Flexibility (z)	Comprehension Gain (z)	Cognitive Risk Index
High Responders	56	31.1	1.18	1.05	0.92	1.12	1.00	0.18
Strategic Improvers	50	27.8	0.42	0.28	0.61	0.54	0.54	0.39
Monitoring-Dependent Learners	44	24.4	−0.22	0.55	−0.22	−0.46	−0.35	0.61
Low Responders	30	16.7	−0.90	−0.72	−0.82	−0.95	−0.88	0.84

Note: Learner-response phenotypes were identified exclusively using baseline planning, monitoring, evaluation, metacognitive knowledge, and strategy flexibility variables. Reading-comprehension gain was not included in the clustering procedure and is presented only as an external post-intervention outcome variable for phenotype characterization.

**Table 5:** Predictive performance and validation metrics of explainable machine-learning models for comprehension-gain prediction.

Model	R^2^	RMSE	MAE	10-Fold Cross-Validation	Calibration Error	Optimization Method
Random Forest	0.56	0.64	0.49	0.53 ± 0.04	0.071	Grid Search
XGBoost	0.61	0.58	0.44	0.59 ± 0.03	0.063	Bayesian Optimization
LightGBM	0.59	0.60	0.46	0.57 ± 0.03	0.067	Bayesian Optimization
Elastic Net	0.47	0.71	0.56	0.45 ± 0.05	0.084	Grid Search

**Table 6:** Transition probability matrix across latent learner states following metacognitive intervention.

From/to	High Responders	Strategic Improvers	Monitoring-Dependent Learners	Low Responders
High Responders	0.67	0.14	0.11	0.08
Strategic Improvers	0.24	0.56	0.13	0.07
Monitoring-Dependent Learners	0.29	0.31	0.28	0.12
Low Responders	0.09	0.17	0.21	0.53

## Data Availability

The datasets generated and analyzed during the current study are available from the corresponding author upon reasonable request. Participant-level data are not publicly available because of confidentiality and ethical restrictions. Participant confidentiality was strictly maintained throughout the study. All datasets were anonymized before analysis, and unique identification codes were assigned to each participant. Personally identifiable information was removed from the analytical dataset and was accessible only to the principal investigators. Electronic data were stored on password-protected devices and used exclusively for research purposes.

## Supplementary Materials

The following supporting information can be downloaded at: https://www.mdpi.com/article/10.3390/jintelligence14070143/s1, Figure S1: Participant selection, eight-week metacognitive intervention, and post hoc computational analysis work-flow; Figure S2: Validation diagnostics of the latent phenotype and explainable machine-learning framework; Figure S3: SHAP dependence and interaction diagnostics for metacognitive predictors of reading comprehension gains; Figure S4: Learner-state redistribution and exploratory simulation diagnostics across learner-response phenotypes; Figure S5: Reinforcement-learning simulation diagnostics and hypothetical adaptive instructional scenarios; Figure S6: Model robustness, sensitivity, and ablation diagnostics of the explainable metacognitive learning framework; Table S1: Demographic, academic, and metacognitive background characteristics of study participants (N = 180); Table S2: Hyperparameter configuration and validation settings for explainable machine-learning models; Table S3: Analytical variables used in each computational stage; Table S4: Instructor monitoring checklist used during the 8-week metacognitive intervention; Table S5: Sensitivity analysis of latent learner-response phenotypes; Table S6: ANOVA and post hoc comparison analyses across latent learner-response phenotypes; Table S7: Bayesian posterior parameter estimates and convergence diagnostics of the adaptive metacognitive intervention framework.
