# Supplementary material for: Adaptive Cognitive Intervention Architecture: An Exploratory Computational Framework for Precision Reading Comprehension in Higher Education
Source: J Intell. 2026 Jul 8;14(7):143. doi: 10.3390/jintelligence14070143 (PMC13413156; doi:10.3390/jintelligence14070143)
Supplement: Supplementary file 1 [file jintelligence-14-00143-s001.zip › jintelligence-4337976-supplementary.pdf]

Adaptive Cognitive Intervention Architecture: An Exploratory Computational Framework for Precision Reading Comprehension in Higher Education

Supplementary Table S1. Demographic, academic, and metacognitive background characteristics of study participants (N = 180).

Variable	Category	n	%
Gender	Male	84	46.7
	Female	96	53.3
Age Group	17–18 years	49	27.2
	19–20 years	78	43.3
	21–22 years	41	22.8
	≥23 years	12	6.7
Academic Level	First Year	52	28.9
	Second Year	61	33.9
	Third Year	43	23.9
	Fourth Year	24	13.3
Weekly Reading Frequency	≤1 session/week	31	17.2
	2–3 sessions/week	82	45.6
	≥4 sessions/week	67	37.2
Average Daily Digital Learning Exposure	<1 hour/day	28	15.6
	1–3 hours/day	96	53.3
	>3 hours/day	56	31.1
Prior Metacognitive Strategy Training	Yes	58	32.2
	No	122	67.8
Baseline Reading Comprehension Level	Low	42	23.3
	Moderate	91	50.6
	High	47	26.1
Baseline Metacognitive Regulation	Low	39	21.7
	Moderate	95	52.8
	High	46	25.5
Preferred Reading Modality	Print-Based	64	35.6
	Digital	78	43.3
	Hybrid	38	21.1
Academic Engagement Level	Low	34	18.9
	Moderate	97	53.9
	High	49	27.2
Intervention Adherence	<70% session completion	26	14.4
	70–89% completion	81	45.0
	≥90% completion	73	40.6

Supplementary Table S2. Hyperparameter configuration and validation settings for explainable machine-learning models.

Model	Hyperparameter	Value
Random Forest	n_estimators	500
	max_depth	8
	min_samples_split	4
	min_samples_leaf	2
	max_features	Sqrt

XGBoost	learning_rate	0.01
	max_depth	5
	subsample	0.80
	colsample_bytree	0.85
	n_estimators	400
	gamma	0.10
LightGBM	num_leaves	31
	learning_rate	0.01
	feature_fraction	0.82
	bagging_fraction	0.80
	n_estimators	450
Elastic Net	alpha	0.15
	l1_ratio	0.60
	max_iter	5000
Cross-Validation Strategy	k-fold validation	10-fold
Optimization Procedure	Hyperparameter tuning	Bayesian optimization + grid search

Supplementary Table S3. Analytical variables used in each computational stage.

**Stage**	**Variables**
Clustering	Planning, Monitoring, Evaluation, Metacognitive Knowledge, Strategy Flexibility
Machine learning	Baseline metacognitive variables, demographics, academic engagement, phenotype membership
Markov	Learner phenotypes
Bayesian	Planning, Monitoring, Evaluation, Metacognitive Knowledge, Strategy Flexibility
Reinforcement learning	Learner states, instructional actions, adaptive rewards

Supplementary Table S4. Instructor monitoring checklist used during the 8-week metacognitive intervention.

**Domain**	**Operational definition**	**Recording method**	**Recording frequency**
Attendance	Presence of participant during the intervention session	Present/Absent	Every session
Classroom participation	Active involvement in discussions and intervention activities	Low/Moderate/High	Every session
Planning strategy use	Evidence of goal setting, previewing texts, and planning reading activities	Yes/No	Every session
Monitoring strategy use	Evidence of self-checking comprehension during reading activities	Yes/No	Every session
Evaluation strategy use	Evidence of reflecting on comprehension outcomes and identifying errors	Yes/No	Every session
Strategy flexibility	Evidence of modifying or changing strategies when encountering difficulties	Yes/No	Every session
Learning progress	Overall progression observed throughout the intervention activities	Low/Moderate/High	Weekly summary

Notes: The instructor monitoring checklist was used solely to document intervention implementation and participant engagement during the intervention sessions. These records were not treated as independent outcome variables in the statistical analyses but were used to verify intervention fidelity and participant involvement.

Supplementary Table S5. Sensitivity analysis of latent learner-response phenotypes.

**Metric**	**Original model**	**Baseline-only model**
Number of clusters	4	4
Silhouette coefficient	0.51	0.49
Davies-Bouldin index	0.74	0.78
Membership agreement (%)	-	87.2

Supplementary Table S6. ANOVA and post hoc comparison analyses across latent learner-response phenotypes.

**Comparison**	**Mean Difference**	**p-value**	**95% Confidence Interval**	**Cohen's d**
High Responders vs Strategic Improvers	0.88	<0.001	0.54 to 1.21	0.93
High Responders vs Monitoring-Dependent	1.58	<0.001	1.19 to 1.96	1.74
High Responders vs Low Responders	2.94	<0.001	2.51 to 3.38	2.63
Strategic Improvers vs Monitoring-Dependent	0.71	0.002	0.29 to 1.07	0.82
Strategic Improvers vs Low Responders	2.08	<0.001	1.66 to 2.47	1.96
Monitoring-Dependent vs Low Responders	1.36	<0.001	0.98 to 1.71	1.41

Overall ANOVA Statistics

**Variable**	**F-value**	**p-value**	**Partial η²**
Reading Comprehension Gain	34.81	<0.001	0.41
Metacognitive Regulation	29.47	<0.001	0.37
Evaluation	31.16	<0.001	0.39
Strategy Flexibility	26.88	<0.001	0.35

Supplementary Table S7. Bayesian posterior parameter estimates and convergence diagnostics of the adaptive metacognitive intervention framework.

**Parameter**	**Posterior Mean**	**SD**	**95% Credible Interval**	**R-hat**
Regulation Effect	0.62	0.09	0.44 to 0.79	1.00
Evaluation Effect	0.57	0.08	0.41 to 0.73	1.00
Monitoring Effect	0.49	0.07	0.35 to 0.63	1.01
Strategy Flexibility Effect	0.44	0.08	0.28 to 0.60	1.00
Metacognitive Knowledge Effect	0.39	0.07	0.24 to 0.53	1.00
Intervention Intensity Effect	0.54	0.10	0.35 to 0.74	1.01
Adaptive Reward Function	0.68	0.11	0.46 to 0.88	1.00
Cognitive Risk Penalty	-0.42	0.08	-0.58 to -0.27	1.00

MCMC Sampling Diagnostics

**Diagnostic Indicator**	**Value**
Number of Chains	4
Iterations per Chain	5000
Warm-up Iterations	1000
Effective Sample Size	>2000
Divergent Transitions	0
Mean Acceptance Probability	0.91

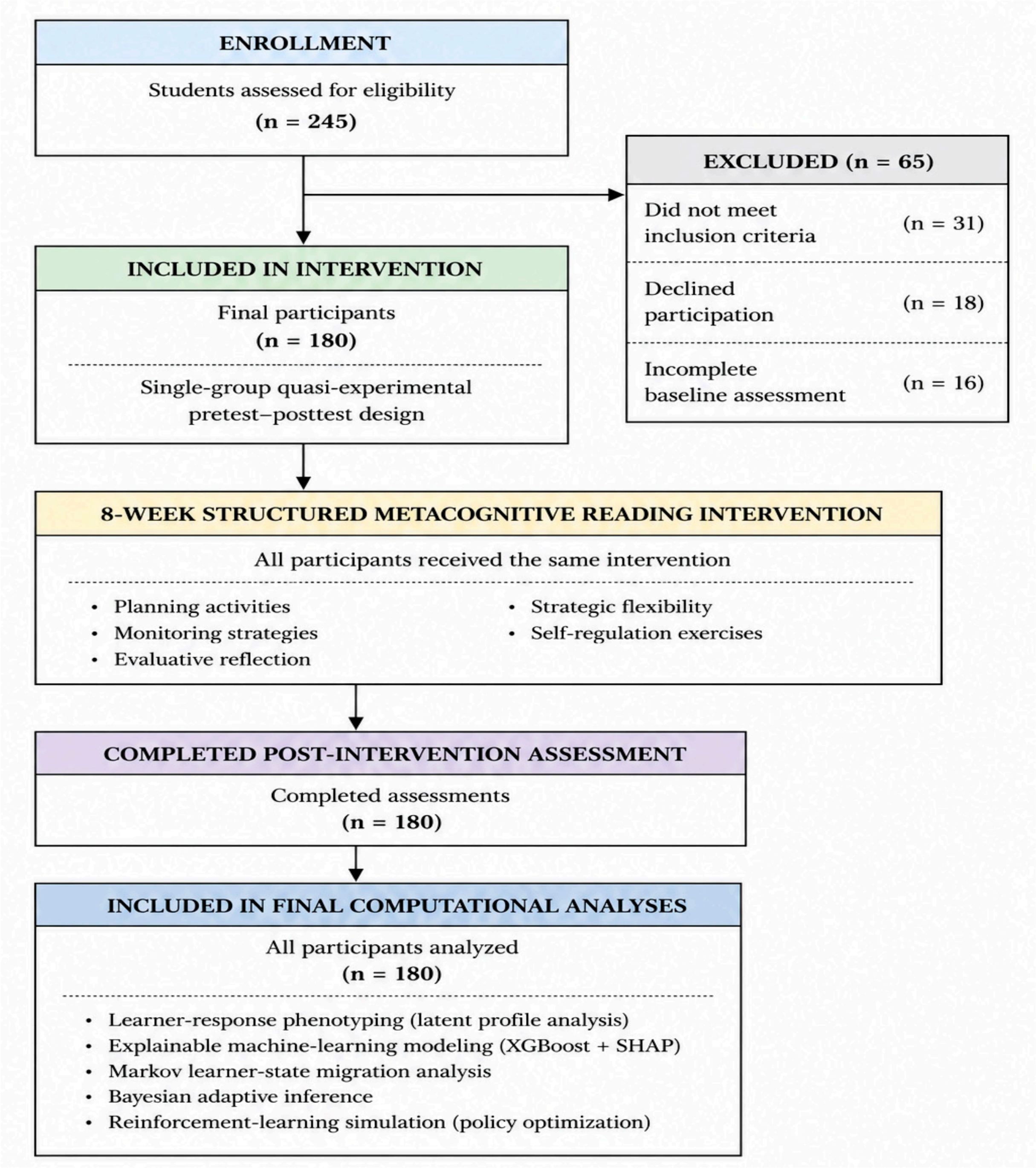

Supplementary Figure S1. Participant selection, eight-week metacognitive intervention, and post hoc computational analysis workflow.

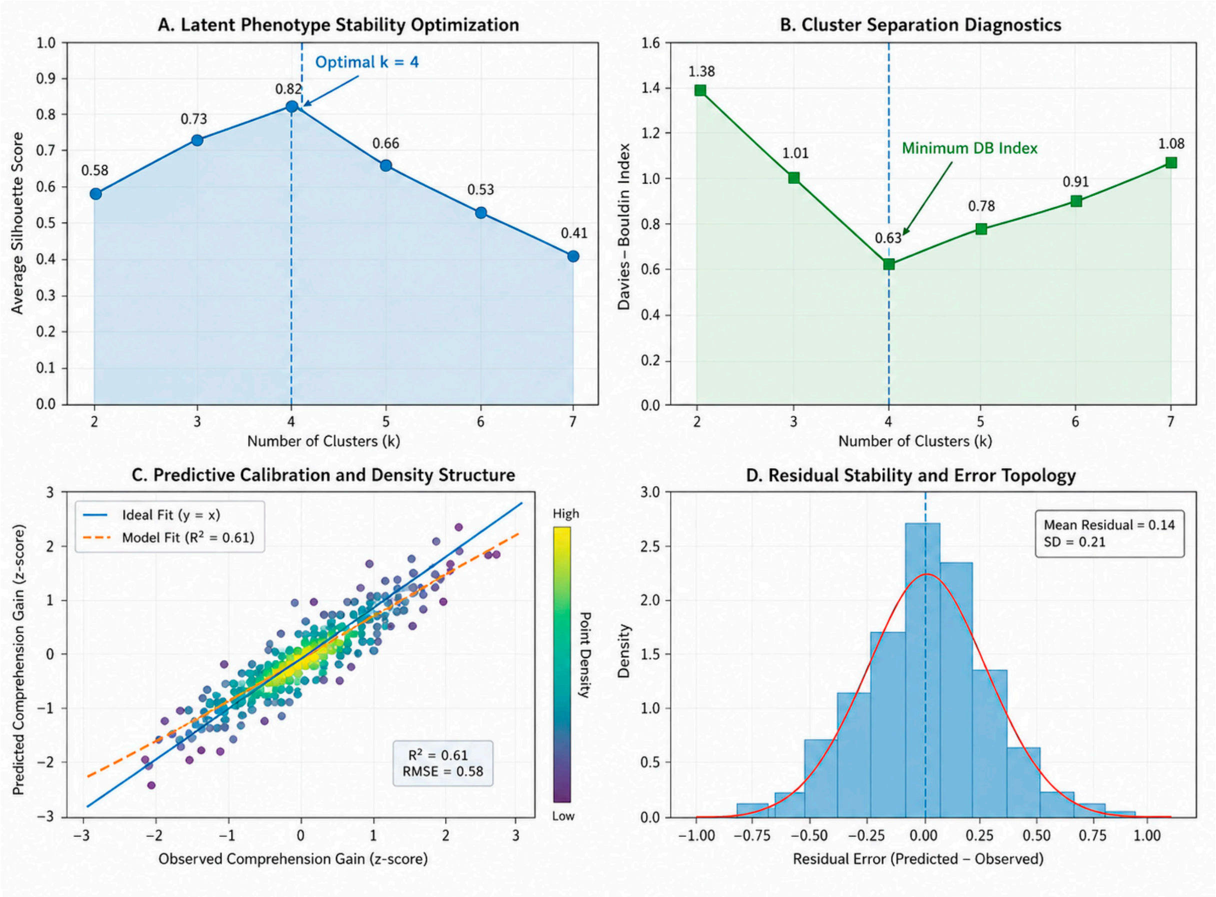

Supplementary Figure S2. Validation diagnostics of the latent phenotype and explainable machine-learning framework.

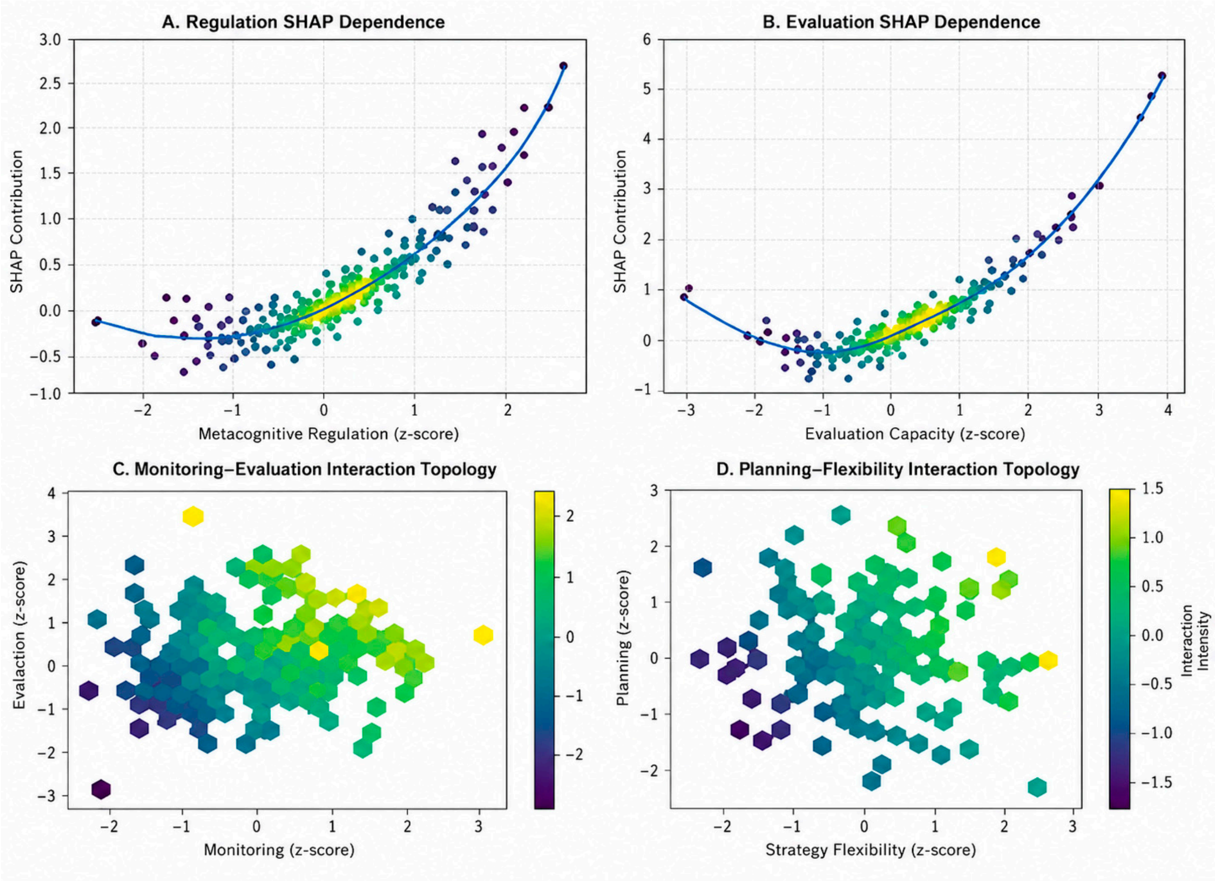

Supplementary Figure S3. SHAP dependence and interaction diagnostics for metacognitive predictors of reading-comprehension gains.

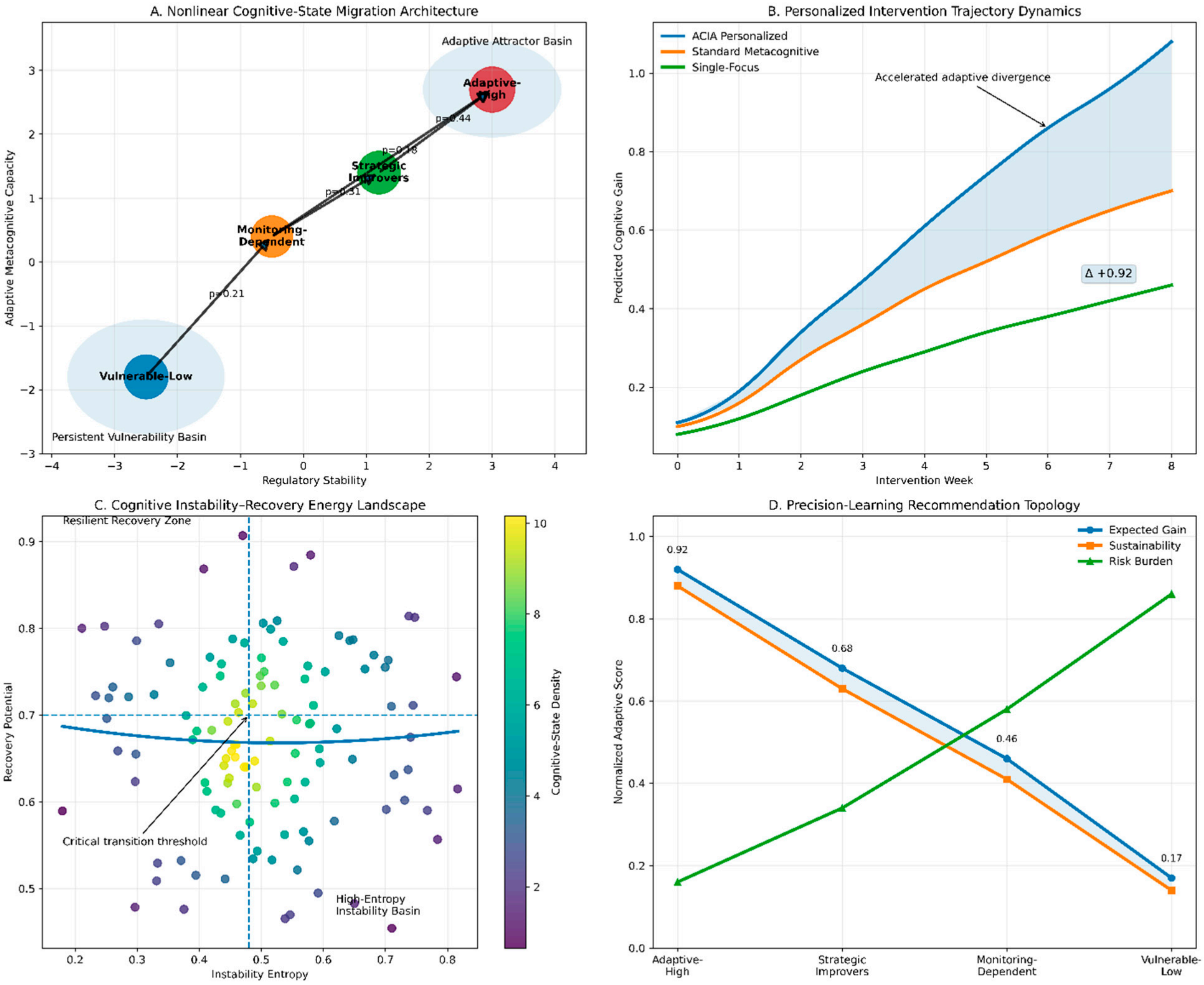

Supplementary Figure S4. Learner-state redistribution and exploratory simulation diagnostics across learner-response phenotypes.

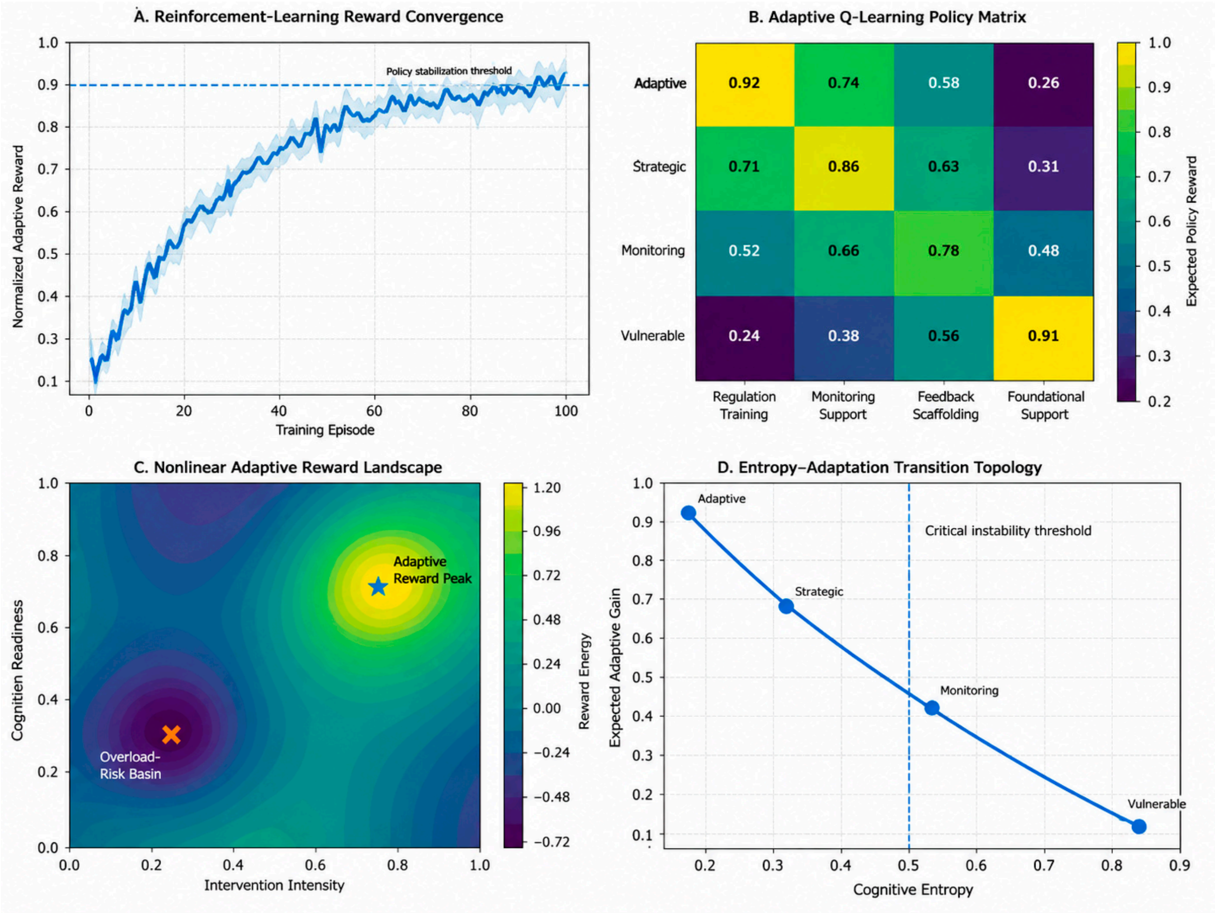

Supplementary Figure S5. Reinforcement-learning simulation diagnostics and hypothetical adaptive instructional scenarios.

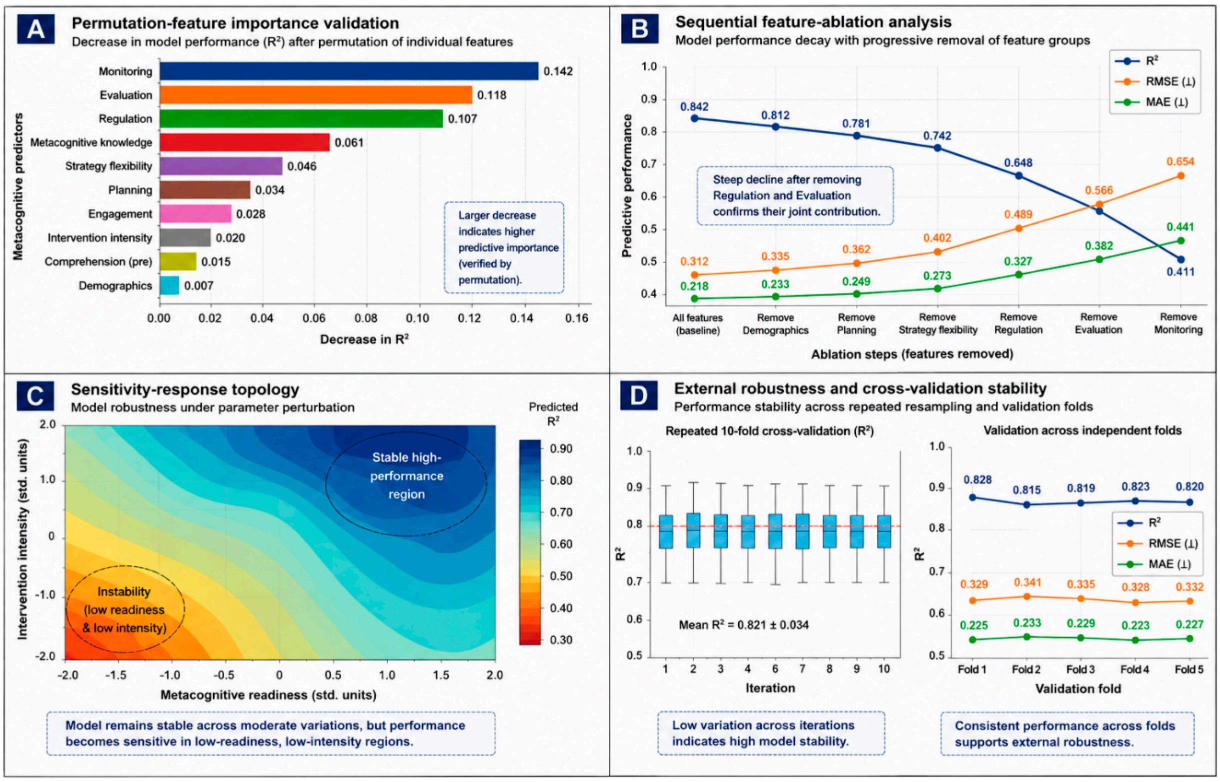

Supplementary Figure S6. Model robustness, sensitivity, and ablation diagnostics of the explainable metacognitive learning framework.
